# Immunogenicity and safety of different dosing schedules of trivalent inactivated influenza vaccine in pregnant women with HIV: a randomised controlled trial

**DOI:** 10.1016/S2352-3018(19)30322-4

**Published:** 2020-01-03

**Authors:** Marta C Nunes, Clare L Cutland, Andrew Moultrie, Stephanie Jones, Justin R Ortiz, Kathleen M Neuzil, Keith P Klugman, Eric A F Simões, Adriana Weinberg, Shabir A Madhi, Maternal Flu Trial Team, A Hugo, P Sithole, L-A Stoltenkamp, Y Abdoola, N van Niekerk, F Treurnicht

**Affiliations:** a Medical Research Council: Respiratory and Meningeal Pathogens Research Unit, School of Pathology, Faculty of Health Sciences, Johannesburg, South Africa; b Department of Science and Technology/National Research Foundation, SARCHI: Vaccine Preventable Diseases, University of the Witwatersrand, Johannesburg, South Africa; c Center for Vaccine Development and Global Health, University of Maryland School of Medicine, Baltimore, MD, USA; d School of Public Health, Center for Global Health, University of Colorado, Aurora, CO, USA; e Department of Pediatric Infectious Diseases, University of Colorado, Aurora, CO, USA; f Department of Pediatrics, Medicine and Pathology, University of Colorado, Aurora, CO, USA

## Abstract

**Background:**

Standard-dose, seasonal, trivalent, inactivated influenza vaccine induces moderate-to-low haemagglutination-inhibition antibody responses in people living with HIV. This study assessed the immunogenicity and safety of different dosing schedules of inactivated influenza vaccine in pregnant women living with HIV in South Africa.

**Methods:**

In this double-blind, randomised, controlled trial, we recruited pregnant women with HIV from seven antenatal clinics in Soweto, South Africa. Pregnant women were eligible if they were aged 18–38 years, infected with HIV, and had an estimated gestational age of 12–36 weeks. Women were randomly assigned (1:1:1), using a computer-generated randomisation list, to receive inactivated influenza vaccine containing 15 μg of each of the three seasonal influenza strains for that year, as a single dose, a double dose, or two single doses 1 month apart. Participants and study personnel were masked to group allocation. Haemagglutination-inhibition antibody responses were measured for all groups in the mothers at enrolment and at 1 month after each vaccine dose, and in the single-dose and double-dose groups within 7 days of birth in the neonates. Immunogenicity analyses only included women with visits 28–35 days apart and infants who were born at least 28 days after maternal immunisation. The primary was seroconversion rate to each of the vaccine strains in the mothers 1 month after completion of the dosing schedule, and the primary safety outcomes were frequency of local and systemic reactions. Safety was assessed in mothers and infants until 24 weeks post partum and analysed in all participants who received at least one dose of vaccine. This study is registered with ClinicalTrials.gov, NCT01527825, and is closed to accrual.

**Findings:**

Between Feb 11, and June 6, 2013, 800 pregnant women living with HIV were enrolled and randomly assigned to the single-dose (n=266), double-dose (n=265), or two-single-doses (n=269) group. In the analysable population, seroconversion rates in mothers 1 month after the final vaccine dose were significantly higher in the double-dose group (n=230; ranging from 29% to 65% for the three vaccine strains) than in the single-dose group (n=230; ranging from 18% to 49%; p≤0·019 for the three vaccine strains), but were similar between the two-single-doses group (n=220; ranging from 23% to 52%) and the single-dose group (p≥0·20 for the three vaccine strains). Safety outcomes were similar in the three groups, except for more injection-site reactions in recipients in the double-dose group.

**Interpretation:**

A regimen of double-dose inactivated influenza vaccine gave slightly greater immunogenicity than did a single-dose regimen in pregnant women living with HIV. However, immunogenicity in the double-dose group was still lower than historical data from the same setting in pregnant women without HIV. More immunogenic vaccines are needed for pregnant women living with HIV to enhance transplacental transfer of vaccine-induced protective antibodies to their newborn infants.

**Funding:**

Bill & Melinda Gates Foundation.

## Introduction

Robust evidence exists to support seasonal influenza vaccination of pregnant women.^1^ Randomised controlled trials have shown the efficacy of seasonal trivalent inactivated influenza vaccine during pregnancy in reducing influenza illness in pregnant women and their infants.^2^, ^3^, ^4^, ^5^ The only randomised controlled trial to date to assess efficacy of influenza vaccination during pregnancy in protecting infants born to women living with HIV was done in South Africa in 2011.^5^ This study, although not powered for efficacy, did not find that newborn infants exposed to HIV had any vaccine-induced protection (vaccine efficacy 26·7%, 95% CI −132·0 to 76·8), possibly due to the modest immune response to inactivated influenza vaccine in pregnant women living with HIV.^5^

In South Africa, after vaccination, pregnant women living with HIV had lower titres of hemagglutination-inhibition antibodies and lower rates of seroconversion (41% *vs* 92%, to at least one vaccine strain) than did those without HIV.^6^ Although transplacental antibody transfer was similar in the women with and without HIV for two of the three vaccine strains conatined in the vaccine, because of the lower concentration of antibodies after vaccination among the women with HIV, their newborn babies had lower haemagglutination-inhibition antibody titres at birth, and were less likely to have haemagglutination-inhibition titres of 1/40 or higher (putative relative correlate of protection), than the newborn babies of women without HIV (range 43–79% of babies exposed to HIV *vs* 82–95% of babies not exposed to HIV for the different vaccine strains).^6^

**Research in context**

**Evidence before this study**

Four randomised clinical trials have shown that vaccination of pregnant women against influenza reduces the risk of influenza illness in their infants during the first 6 months of life. However, the only trial that assessed vaccine efficacy among infants born to women living with HIV in South Africa did not detect a protective effect in this group. The lack of vaccine efficacy in infants exposed to HIV might be related to the reduced immune response to vaccination in pregnant women living with HIV. We searched PubMed for publications in English up until May 1, 2019, with no start date restriction using the terms “maternal influenza vaccination HIV”, “maternal influenza immunization HIV”, “pregnancy influenza vaccination HIV”, and “pregnancy influenza immunization HIV”. We found only one study that described immunogenicity in pregnant women with HIV and the antibody levels of their infants after two doses of inactivated A/H1N1/2009 pandemic monovalent vaccine (two doses of 30 μg) during pregnancy. That study reported that a two double doses regimen was moderately immunogenic and that seroprotective titres were present in 67% of mothers and 65% of infants at delivery. After the second dose, only a slight improvement in seroprotection was seen. We did the present analysis to assess if increasing the vaccine antigen content or administration of a second dose of vaccine would improve the immunogenicity of trivalent inactivated influenza vaccine in pregnant women living with HIV and the protection of their infants against influenza infection compared with a standard single dose of vaccination.

**Added value of this study**

To our knowledge, this is the first randomised clinical trial that has measured the immunogenicity, safety, and efficacy of three different schedules for trivalent inactivated influenza vaccine dosing in pregnant women living with HIV. We found that a double dose of inactivated influenza vaccine had moderately improved immunogenicity in pregnant women living with HIV, although no difference was observed in the incidence of symptomatic influenza in the women or their infants.

**Implications of all the available evidence**

Despite lower antibody responses to influenza vaccination in individuals living with HIV than in previous studies in those without HIV, we found that vaccine efficacy supports the continued vaccination of this vulnerable population. However, continued research is needed to find improved influenza vaccines for pregnant women living with HIV to improve protection of their infants.

The randomised controlled trials in South Africa also revealed a higher incidence of PCR-confirmed influenza in women with HIV than in women without, both in those given placebo (17·0% *vs* 3·6%) and those given vaccine (7·0% *vs* 1·8%).^5^ However, overall vaccine efficacy against PCR-confirmed influenza was similar in women living with HIV (57·7%) and those without HIV (50·4%).^5^ Several other studies have also reported lower haemagglutination-inhibition antibody responses to inactivated influenza vaccine in individuals living with HIV than in the general population, although with some conflicting results.^7^, ^8^, ^9^, ^10^

Considering the decreased immunogenicity of standard-dose influenza vaccines in individuals living with HIV, different vaccines or vaccination strategies, such as use of adjuvants, an extra dose of vaccine, or a high-dose inactivated influenza vaccine containing 60 μg of antigen per strain, have been assessed to improve immune response to seasonal vaccines and the 2009 pandemic A/H1N1 monovalent vaccines.^11^, ^12^, ^13^, ^14^, ^15^, ^16^, ^17^ Adjuvanted vaccines and high-dose inactivated influenza vaccines elicited increased haemagglutination-inhibition titres and improved seroresponse rates in individuals living with HIV compared with standard doses, but responses were still inferior compared with adults without HIV receiving standard doses of vaccine.^11^, ^12^ Furthermore, a second vaccine dose did not consistently increase haemagglutination-inhibition antibody responses.^11^, ^13^, ^14^, ^15^, ^18^

We hypothesised that increasing the antigen content of a vaccine or administration of a second dose of vaccine would improve the immunogenicity of inactivated influenza vaccine in pregnant women with HIV and the protection of their infants against PCR-confirmed influenza compared with standard-dose inactivated influenza vaccine.

## Methods

### Study design and participants

In this double-blind, randomised, controlled trial, study staff screened pregnant women with documented HIV-1 infection for study participation at seven antenatal clinics in Soweto, South Africa. Soweto is an urban low-income setting with a predominantly black African population of an estimated 1·2 million people located in the outskirts of Johannesburg. HIV prevalence among pregnant women in Soweto has remained stable at 29% since 2009.^19^

Pregnant women were eligible if they were aged 18–38 years, had an estimated gestational age of 12–36 weeks, and were able to understand and comply with the study protocol. Exclusion criteria included any features of WHO clinical category 3 or 4 of AIDS, having been given any other inactivated influenza vaccine during the current influenza season or received any live licensed vaccine in the past 28 days or an inactivated licensed vaccine in the past 14 days before the study. Full exclusion criteria are in the appendix (p 2). We enrolled participants before the anticipated onset of the influenza season in South Africa. The 2013 South African influenza epidemic period was from April 22 to Oct 13.^20^

The study was approved by the Human Research Ethics Committee of the University of the Witwatersrand (111114) and done in accordance with Good Clinical Practice guidelines. Mothers provided written informed consent for themselves and their infants. The study protocol is available in the appendix (pp 10–70).

### Randomisation and masking

Participants were randomly assigned (1:1:1) to receive treatment with a single dose of inactivated influenza vaccine followed by placebo (single-dose group), a double dose of inactivated influenza vaccine followed by placebo (double-dose group), or two single doses of inactivated influenza vaccine (two-single-doses group). The second injection for each group was given 1 month after the first injection.

An unmasked study statistician created computer-generated randomisation lists in blocks of 30, with ten blocks in each group (single-dose group, double-dose group, and two-single-doses group), with assignment of consecutive four-digit study numbers to the randomisation list. The randomisation forms were preprinted with the four-digit study numbers and an alphabetical and colour code for vaccine and placebo. Participants were allocated consecutive study numbers in order of enrolment at each study clinic. Participants and study personnel were masked to group allocation. Study medication was prepared by an unmasked pharmacist in syringes filled with either saline or inactivated influenza vaccine as per randomisation and labelled as dominant and non-dominant, and study nurses who enrolled the participants selected syringes matching the codes reflected in the randomisation form (appendix p 3). The pharmacist and study statistician were not involved in participant follow up.

### Procedures

Participants were given a commercially produced influenza vaccine (Vaxigrip; Sanofi-Pasteur, Lyon, France) that contained 15 μg of A/California/7/2009 (A/H1N1pdm09), A/Victoria/361/2011 (A/H3N2), and B/Wisconsin/1/2010 (Yamagata-lineage), as recommended for the southern hemisphere in 2013. Vaxigrip was approved in South Africa for use as a single dose in adults. The study pharmacist prepared inactivated influenza vaccine or sterile 0·9% normal saline solution (placebo) in identical syringes; the two preparations were visually indistinguishable. At enrolment (visit 1), all participants received two injections: inactivated influenza vaccine and placebo were given to the single-dose and two-single-doses groups, one in each arm, with inactivated influenza vaccine given in the non-dominant arm; and one dose of inactivated influenza vaccine was given in each arm of the participants in the double-dose group (ie, 30 μg of antigen per strain). 1 month later (visit 2), all participants received a single injection on their non-dominant arm, with the single-dose and double-dose groups both given placebo, and the two-single-doses group given another dose of inactivated influenza vaccine.

Participant follow-up was done at the Respiratory and Meningeal Pathogens Research Unit, Soweto, South Africa. We tested immune responses on plasma samples collected from the women immediately before the first injection at visit 1, 1 month after the first injection and immediately before the second injection at visit 2, and 1 month after the second injection at visit 3. We also collected blood within 7 days of birth from the women and their neonates. After review of maternal haemagglutination-inhibition antibody titres, and that no differences were detected between the single-dose and two-single-doses groups, we made a post-hoc decision to analyse only infant blood collected from infants in the single-dose and double-dose groups. Haemagglutination-inhibition antibodies were measured as described previously.^5^, ^21^

We did weekly active surveillance by home visit or telephone call from the time of enrolment through to 24 weeks post partum in mothers and infants for influenza-like illness. The criteria for diagnosing influenza-like illness in the women were onset in the past 7 days of symptoms including: a fever of 38·0°C or higher on oral measurement or history of chills, rigors, or feeling feverish; presence of cough, sore throat, or pharyngitis; presence of myalgia, arthralgia, or headache; or presence of dyspnoea, breathing difficulty, or chest pain when breathing. Criteria used for diagnosing influenza-like illness in infants were any of the following: axillary temperature of 37·8°C or higher or mother's perception that an infant was feverish, or both, without evidence of a non-respiratory localised source, coupled with at least one sign or symptom of acute respiratory illness in the past 72 h; or at least two signs or symptoms of acute respiratory illness in the past 72 h. Signs or symptoms of acute respiratory illness were respiratory rate of 60 or more breaths per min in infants aged 0–2 months and 50 or more breaths per min in infants aged 2–6 months and difficulty breathing reported by the mother, cough, wheezing, runny or congested nose, cyanosis or oxygen saturation below 90% on room air, chest wall in-drawing, grunting on expiration, and pus draining from either ear. Women and infants identified as having influenza-like illness during surveillance visits had respiratory secretions collected within 72 h of illness identification that were tested by influenza PCR. We also investigated by influenza PCR, participants who were admitted to hospital and who had unsolicited study clinic visits due to respiratory illness. Details on sample collection, influenza diagnostic and subtyping by PCR have been described previously.^5^

For routine assessment of reactogenicity and safety, the investigator (usually study nurse) observed each participant for 30 min after each injection to monitor immediate adverse events. Additionally, participants were provided with a diary card to document solicited local and systemic reactions for 7 days after each vaccination visit. For injection site reactions, severe reactions were defined as follows: severe tenderness as severe pain in the injected limb that increases when moved or when the movement was reduced, severe redness as redness in an area of 100 mm or larger in size, severe swelling as swelling in an area of 100 mm or larger in size, severe hardness as a large lump being felt wider than half the arm width, severe bruising as a bruise larger than 25 mm in size, and severe itching as itching that requires soothing cream. For systemic reactions, severe reactions were defined as severe weakness or tiredness as being unable to do normal activities during the day, severe headache as a headache requiring medication and being unable to do normal activities during the day; severe fever as an axillary temperature of 39·4°C or higher, and severe joint or muscle pain as severe aching that required medication and that restricted activity. Serious adverse events were collected throughout the study until 24 weeks post partum in women and infants.

### Outcomes

The coprimary study objectives were to assess the relative immunogenicity of a double dose and two single doses of inactivated influenza vaccine compared with a single dose of inactivated influenza vaccine in pregnant women living with HIV to each of the three vaccine strains, and to assess the relative safety of the three dosing schedules. The primary outcome was the seroconversion rate to each of the vaccine strains in mothers 1 month after completion of the dosing schedule.

Secondary objectives reported here were to compare the proportion of neonates born to women in the double-dose group with haemagglutination-inhibition titres of 1/40 or higher at age 7 days or younger (individually for each of the three vaccine strains) with the proportion in the single-dose group, to assess the relative efficacy of the double-dose and two-single-doses regimens compared with the single-dose regimen against PCR-confirmed influenza among the women and their infants until 24 weeks post partum. Additional secondary objectives (appendix p 3) will be reported in the second half of 2020.

Criteria for interpreting haemagglutination-inhibition results included titres of 1/40 or higher as putative measures of relative seroprotection and seroconversion if a four-fold or higher titre increase occurred from baseline to after vaccination with haemagglutination-inhibition antibody titres of 1/40 or higher after vaccination.

Primary safety outcomes were the frequency of solicited local and systemic reactions after a single vaccine dose, a double vaccine dose, or two single doses.

### Statistical analysis

We powered the sample size at 80% with a two-tailed significance level of 0·05 to detect a 30% or higher difference in the proportion of pregnant women living with HIV who seroconverted in the double-dose group or the two-single-doses group, compared with the single-dose group. On the basis of results from our previous study on immune responses to a single dose of inactivated influenza vaccine^5^, we estimated that approximately 44% of women in the single-dose group would have haemagglutination-inhibition antibody titres of 1/40 or higher and a four-fold or higher increase in titres to the least immunogenic strain in the vaccine. The sample size required was 789 women (ie, 263 in each group).

We only included women in the immunogenicity analysis if their vaccination visits were 28–35 days apart and if blood samples were collected 28–35 days after the second injection for those in the two-single-doses group. We only included infants in the immunogenicity analysis if blood samples were collected in the first 7 days of life and if they were born at least 28 days after maternal immunisation. We compared proportions with χ^2^ or Fisher's exact tests and demographic continuous variables with Student's *t* test or the Mann-Whitney test. We estimated geometric mean titres and corresponding 95% CIs by use of logarithmic transformation and compared these between study groups using Student's *t* test. We calculated relative vaccine efficacy against first episode of PCR-confirmed influenza in the intention-to-treat population with the formula (1–I_sg_)/I_SD_, where I_sg_ is the incidence of cases in the double-dose or two-single-doses group, and I_SD_ is the incidence of cases in the single-dose group, and we calculated 95% CIs and we tested between-group differences.

The safety analysis set comprised participants who received at least one dose of vaccine. After the first vaccination, we compared the reactions of women who had been given a single dose of vaccine (the single-dose and two-single-doses groups) with those who had been given a double dose (double-dose group), and after the second vaccination we compared women who had received placebo in the single-dose group only with those who had been given another dose of vaccine (the two-single-doses group). We present safety data as incidences with 95% CIs. We did descriptive analyses for the number of maternal, infant, and fetal deaths, and maternal and infant admissions to hospital.

We considered p values of less than 0·05 to be significant. We collected and managed study data using Research Electronic Data Capture (REDCap).^22^ We did all analyses using STATA version 12.1 (College Station, TX, USA). The study was registered at ClinicalTrial.gov, NCT01527825.

### Role of the funding source

The funder of the study had no role in the study design, data collection, data analysis, data interpretation, or writing of the report. The corresponding author had full access to all the data in the study and had final responsibility for the decision to submit for publication.

## Results

Between Feb 11, and June 6, 2013, we enrolled 800 black African pregnant women living with HIV who were randomly assigned to the single-dose group (n=266), double-dose group (n=265), or two-single-doses group (n=269; figure). Delivery outcomes were established for 774 (97%) of 800 women, including 30 twin pregnancies and one woman in the single-dose group who had a therapeutically indicated medical termination of pregnancy at 25 weeks of gestation (75 days after enrolment). The first infant in the study was born on Feb 22, 2013. 782 livebirths occurred, 136 (17%) of 781 were preterm births (one baby had missing information), 155 (20%) of 779 neonates had a birthweight of less than 2500 g, and eight (1%) of 681 were infected with HIV. No differences were observed between the three study groups in demographic characteristics, pregnancy outcomes, and birth characteristics of their neonates (table 1). The mean time between the first injection and delivery was 99·6 days (SD 44·8); 145 (19%) women delivered fewer than 28 days after the second injection visit, including 30 who delivered fewer than 28 days after the first injection visit.

**Figure:**
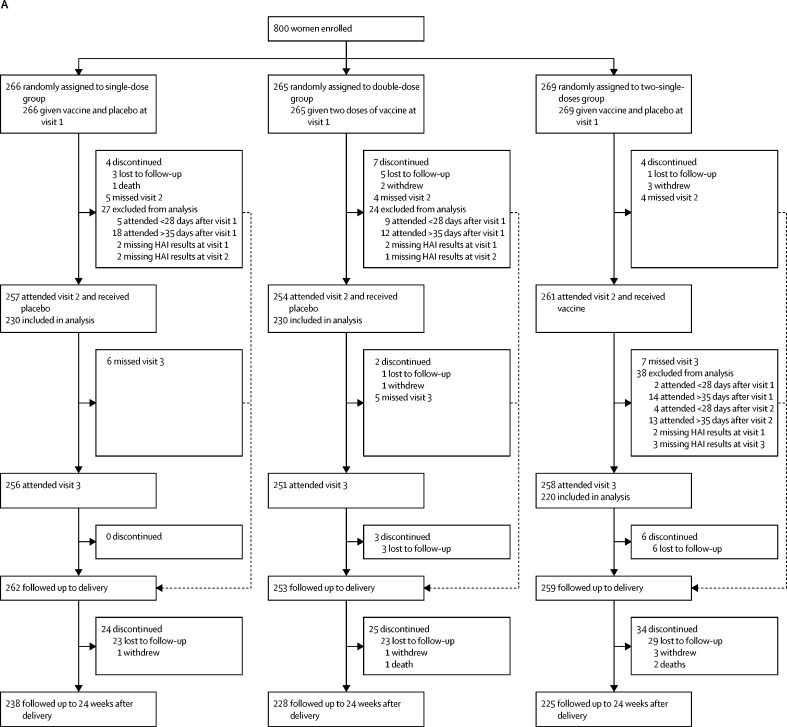

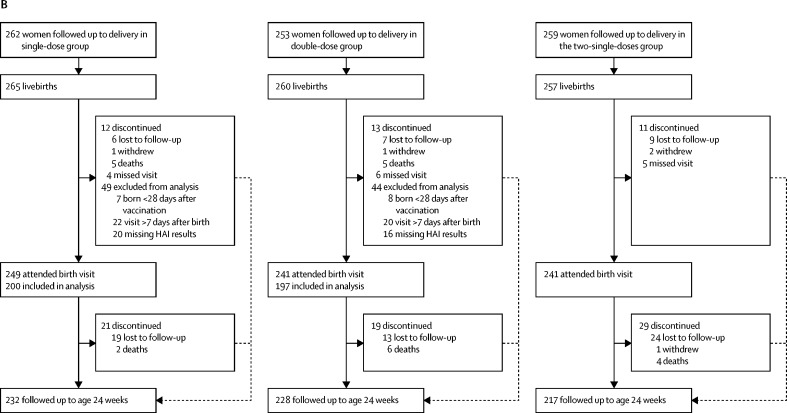
Trial profile for mothers (A) and their infants (B) Visit 1 is the enrolment and first injection visit, visit 2 is the second injection visit, and visit 3 is the visit 1 month after the second injection. HAI=hemagglutination-inhibition antibody.

**Table 1:** Maternal demographic and clinical characteristics at time of enrolment and delivery outcomes

		**Single-dose group (n=266)**	**Double-dose group (n=265)**	**Two-single-doses group (n=269)**	**Total population (n=800)**
**Demographic and clinical characteristics**					
Age, years		28·8 (5·0)	29·0 (4·8)	28·4 (4·9)	28·7 (4·9)
Gestational age at enrolment, weeks		23·4 (6·0)	24·2 (6·1)	23·8 (5·6)	23·8 (5·9)
Primigravida women		30 (11%)	40 (15%)	31 (12%)	101 (13%)
CD4 count within 2 weeks of enrolment					
	n	257	255	263	775
	Mean, cells per μL	458·7 (260·5)	463·9 (256·5)	454·4 (227·6)	459·0 (248·2)
	<350 cells per μL	99/257 (39%)	91/255 (36%)	98/263 (37%)	288/775 (37%)
HIV viral load within 2 weeks of enrolment					
	n	245	243	249	737
	Median, copies per mL*	4918 (475–34 863)	7298 (511–32 360)	5152 (711–28 486)	6066 (524–33 044)
	<40 copies per mL	65/245 (27%)	59/243 (24%)	72/249 (29%)	196/737 (27%)
Antiretroviral therapy at enrolment		248/263 (94%)	250/262 (95%)	256/267 (96%)	754/792 (95%)
	On antiretroviral therapy for ≥30 days	129/255 (51%)	126/255 (49%)	143/265 (54%)	398/775 (51%)
**Delivery outcomes**					
Known delivery outcome		262 (98%)†	253 (95%)	259 (96%)	774 (97%)†
Number of twin pregnancies		9/262 (3%)	12/253 (5%)	9/259 (3%)	30/774 (4%)
Days between first injection and delivery‡		102·9 (45·9)	96·9 (45·6)	98·8 (42·8)	99·6 (44·8)
Delivered <28 days after first injection‡		9/262 (3%)	8/253 (3%)	13/259 (5%)	30/774 (4%)
Delivered <28 days after second injection‡		47/262 (18%)	54/253 (21%)	44/259 (17%)	145/774 (19%)
Birth outcomes known		270†§	265	268¶	803†§
Fetal deaths		5/270 (2%)	5/265 (2%)	11/268 (4%)	21/803 (3%)
Livebirths		265	260	257	782
Births <37 weeks of gestation||		48/264 (18%)	38/260 (15%)	50/257 (19%)	136/781 (17%)
Birthweight, kg||		2·9 (0·6)	2·9 (0·6)	2·9 (0·6)	2·9 (0·6)
Neonates with <2500 g birthweight||		52/263 (20%)	53/260 (20%)	50/256 (20%)	155/779 (20%)
Infants exclusively breastfed at birth visit		103/247 (42%)	103/235 (44%)	96/239 (40%)	302/721 (42%)

Data are mean (SD), median (IQR), n (%), or n/N (%).

* Excludes participants with a viral load of <40 copies per mL HIV.

† Excluding one woman who died while pregnant.

‡ Based on women with known delivery outcomes.

§ Excluding one woman who had a therapeutically indicated medical termination of pregnancy.

¶ 14 birth outcomes occurred before second vaccination visit.

|| Based on livebirths.

Before the first injection, haemagglutination-inhibition antibody geometric mean titres were similar across the three study groups for the three vaccine strains. Also, similar proportions of women in all groups had haemagglutination-inhibition titres of 1/40 or higher for any of the three vaccine strains (Table 2, Table 3), except for a higher proportion of women in the two-single-doses group for A/H3N2 (p=0·023).

**Table 2:** Immune responses in pregnant women living with HIV and antibody levels in their infants, in the single-dose and double-dose groups

		**A/H1N1pdm09**			**A/H3N2**			**B/Yamagata**		
		Single-dose group	Double-dose group	p value	Single-dose group	Double-dose group	p value	Single-dose group	Double-dose group	p value
Mothers*		230	230	··	230	230	··	230	230	··
	HAI GMTs at baseline	9·6 (8·5–10·9)	9·5 (8·4–10·8)	0·92	11·8 (10·5–13·3)	13·3 (11·7–15·1)	0·19	5·9 (5·6–6·2)	6·1 (5·7–6·5)	0·39
	HAI GMTs at 28–35 days after visit 1†	39·5 (33·1–47·1)	60·8 (51·2–72·2)	0·001	37·8 (32·3–44·1)	52·7 (45·4–61·1)	0·003	13·0 (11·6–14·5)	16·6 (14·7–18·8)	0·004
	HAI ≥1/40 at baseline	33 (14%)	29 (13%)	0·59	34 (15%)	50 (22%)	0·053	4 (2%)	4 (2%)	1·0
	HAI ≥1/40 at 28–35 days after visit 1	142 (62%)	171 (74%)	0·004	131 (57%)	163 (71%)	0·002	46 (20%)	71 (31%)	0·007
	Seroconversion at 28–35 days after visit 1	113 (49%)	150 (65%)	<0·001	95 (41%)	120 (52%)	0·019	41 (18%)	67 (29%)	0·004
Infants overall‡		200	197	··	200	197	··	200	197	··
	HAI GMTs at age ≤7 days	29·7 (24·7–35·5)	38·5 (32·1–46·1)	0·046	23·3 (19·7–27·5)	26·2 (22·3–30·9)	0·32	13·0 (11·7–14·4)	14·4 (12·8–16·2)	0·20
	HAI ≥1/40 at age ≤7 days	99 (50%)	118 (60%)	0·037	77 (39%)	90 (46%)	0·15	32 (16%)	37 (19%)	0·47

Data are n, n (%), or GMTs with 95% CI in parentheses. GMTs=geometric means titres. HAI=haemagglutination inhibition antibody.

* Only women who attended the visits within the protocol-defined time periods were included.

† GMTs significantly higher than at baseline for all comparisons.

‡ Only infants born ≥28 days after their mothers had been vaccinated and with blood sample collected in the first 7 days of life were included.

**Table 3:** Immune responses of pregnant women living with HIV in the single-dose and two-single-doses groups

	**A/H1N1pdm09**			**A/H3N2**			**B/Yamagata**		
	Single-dose group (n=230)	Two-single-doses group (n=220)	p value	Single-dose group (n=230)	Two-single-doses group (n=220)	p value	Single-dose group (n=230)	Two-single-doses group (n=220)	p value
HAI GMTs at baseline	9·6 (8·5–10·9)	9·5 (8·4–10·8)	0·76	11·8 (10·5–13·3)	12·5 (11·0–14·2)	0·99	5·9 (5·6–6·2)	5·9 (5·6–6·2)	0·94
HAI GMTs at 28–35 days after completion of full vaccine series*†	39·5 (33·1–47·1)	46·7 (39·7–54·9)	0·17	37·8 (32·3–44·1)	44·1 (37·5–51·9)	0·18	13·0 (11·6–14·5)	15·5 (13·9–17·3)	0·029
HAI antibody ≥1/40 at baseline	33 (14%)	31 (14%)	0·94	34 (15%)	51 (23%)	0·023	4 (2%)	2 (1%)	0·59
HAI antibody ≥1/40 at 28–35 days after completion of full vaccine series†	142 (62%)	148 (67%)	0·22	131 (57%)	145 (66%)	0·051	46 (20%)	54 (25%)	0·25
Seroconversion at 28–35 days after first vaccination†	113 (49%)	115 (52%)	0·51	95 (41%)	103 (47%)	0·24	41 (18%)	50 (23%)	0·20

Data are n (%) and GMTs with 95% CI in parentheses. Only women who attended the visits within the protocol-defined time periods are included. GMTs=geometric means titres. HAI=hemagglutination inhibition antibody.

* GMTs significantly higher than at baseline for all comparisons.

† For single-dose group assessed at 28–35 days after first injection; for two-single-doses group assessed at 28–35 days after second injection.

1 month after the first injection, haemagglutination-inhibition antibody geometric mean titres increased in women in both the single-dose and double-dose groups but were higher in those in the double-dose group than in those in the single-dose group for all three strains (table 2). Similarly, 1 month after vaccination, seroconversion rates were higher in the double-dose group than in the single-dose group for all three strains (table 2). Furthermore, a higher proportion of women in the double-dose group had haemagglutination-inhibition titres of 1/40 or higher to each strain than did in the single-dose group (p≤0·019 for all comparisons; table 2).

200 infants born to mothers into the single-dose group and 197 infants born to mothers in the double-dose group at least 28 days after vaccination had blood samples collected within the first 7 days of life, with the mean time from birth to blood collection in infants being 3·3 days (SD 1·9) in both groups. We made a post-hoc decision to not analyse blood samples from infants in the two-single-doses group. Infants born to mothers in the double-dose group had higher haemagglutination-inhibition antibody geometric mean titres (p=0·046) and a higher proportion had titres of 1/40 or higher for A/H1N1pdm09 than did those born to mothers in the single-dose group (p=0·037). We saw no significant differences in haemagglutination-inhibition antibody geometric mean titres or the proportion of infants with titres of 1/40 or higher between the single-dose group and the double-dose group for A/H3N2 and B/Yamagata (table 2). Similar results were obtained when the 41 eligible preterm infants were excluded in a post-hoc analysis, except that the significant differences for A/H1N1pdm09 were no longer detected (data not shown). Additionally, we saw no difference in geometric mean titres and the proportion of infants with haemagglutination-inhibition antibody titres of 1/40 or higher between preterm and term infants overall and by study group (data not shown).

In mothers, haemagglutination-inhibition geometric mean titres were similar 1 month after the completion of the vaccination series between the single-dose and two-single-doses groups, except for higher geometric mean titres for B/Yamagata after the second dose in the two-single-doses group (table 3). 1 month after completion of full vaccination series, we saw no differences in the proportion of women with titres of 1/40 or higher or who had seroconverted between the single-dose and two-single-doses groups for any of the three vaccine strains (table 3). Notably, we saw no increase in haemagglutination-inhibition antibody geometric mean titres or the proportion of women with titres of 1/40 or higher 1 month after the second dose of vaccine in the two-single-doses group compared with 1 month after the first dose in this group (appendix p 5).

65 (8%) of 800 women had at least one episode of PCR-confirmed influenza, with two women, both in the single-dose group, having two distinct episodes by A/H1N1pdm09 and B/Yamagata more than 2 months apart. 42 PCR-confirmed influenza episodes were detected during pregnancy, with one woman having two episodes while pregnant. PCR-confirmed A/H1N1pdm09 was detected in 34 women, PCR-confirmed A/H3N2 in 22 women, and PCR-confirmed B/Yamagata in 12 women, including one woman in whom both A/H3N2 and B/Yamagata were co-detected. Three women, one in each study group, had PCR-confirmed episodes that were detected fewer than 14 days after the first vaccination visit; and an additional four women in the two-single-doses group had a PCR-confirmed influenza episode either before (n=2) or fewer than 14 days after (n=2) the second dose of vaccine. We saw no differences in the incidence of first episode of PCR-confirmed influenza between women in the single-dose group compared with the double-dose group (p=0·88) or the two-single-doses group (p=0·67) overall or by individual influenza strain (table 4).

**Table 4:** Relative efficacy of a double dose or two single doses compared with a single dose of vaccine against first episode of PCR-confirmed influenza in HIV-infected pregnant women and their infants until 24 weeks after birth

		**Single-dose group**	**Double-dose group**	**Relative vaccine efficacy**	**p value**	**Two-single-doses group**	**Relative vaccine efficacy**	**p value**
Mothers		266	265	··	··	269	··	··
	PCR-confirmed influenza	21 (8%)	20 (8%)	4·4% (−72·2 to 46·9)	0·88	24 (9%)	–13·0% (−98·0 to 35·5)	0·67
	PCR-confirmed A/H1N1pdm09	14 (5%)	8 (3%)	42·6% (−34·4 to 75·6)	0·19	12 (4%)	13·6% (−83·2 to 59·3)	0·70
	PCR-confirmed A/H3N2	4 (2%)	8 (3%)	–100·8% (−558·6 to 38·8)	0·26	10 (4%)	–146·3% (−675·4 to 21·8)	0·17
	PCR-confirmed B/Yamagata	5 (2%)	5 (2%)	–0·4% (−242·7 to 70·6)	0·99	2 (1%)	60·4% (−102·1 to 92·3)	0·25
Infants		265	260	··	··	257	··	··
	PCR-confirmed influenza	6 (2%)	5 (2%)	15·1% (−174·9 to 73·8)	0·99	10 (4%)	–71·9% (−366·0 to 36·6)	0·28

Data are n, n (%), or relative vaccine efficacy compared with single-dose group, with 95% CI in parentheses. Among mothers, the overall number of cases of PCR-confirmed influenza are lower than the sum of the individual strains because one woman in the double-dose group had a double infection with B/Yamagata and A/H3N2 and two women in the single-dose group had two different PCR-confirmed influenza infections, both women with A/H1N1pdm09 and B/Yamagata.

23 PCR-confirmed episodes of influenza (seven of A/H1N1pdm09, five of A/H3N2, ten of B/Yamagata, and one B/Victoria) were recorded among 21 infants. Two infants in the two-single-doses group had two distinct episodes 2 or more months apart (one infant was infected by A/H3N2 and B/Yamagata and the other had two episodes of B/Yamagata infection at age 45 days and 94 days). We saw no differences in the incidence of PCR-confirmed influenza among infants in the single-dose group compared with the double-dose group (p=0·99) or the two-single-doses group (p=0·28; table 4).

The incidence of one or more local solicited reaction was greater in the double-dose group (47·*8*% in the non-dominant arm and 38·*8*% in the dominant arm) than in the single-dose and two-single-doses groups (38·1% non-dominant arm and 26*·7*% dominant arm; p≤0·010 for both), but we saw no difference in the proportion who had severe local reactions (table 5). However, women who were given a single dose of vaccine had a higher frequency of at least one severe solicited systemic reaction than did those given a double dose (p=0·022), mainly headache and weakness or tiredness (table 5). After the second injection, we saw no differences in solicited local and systemic reactions between women in the single-dose group and the two-single-doses group (table 5).

**Table 5:** Solicited local and systemic reactions during the first week after the first dose of vaccine and the second dose of vaccine or placebo in pregnant women living with HIV

		**First week after first vaccination**			**First week after second vaccination**		
		Single-dose and two-single-doses groups (n=517)	Double-dose group (n=255)	p value	Single-dose group (placebo; n=240)	Two-single-doses group (n=238)	p value
**Non-dominant arm local injection-site reactions**							
Tenderness							
	Any	24·6% (20·9–28·5)	28·3% (22·8–34·2)	0·27	15·0% (10·7–20·2)	18·5% (13·8–24·0)	0·31
	Severe	2·7% (1·5–4·5)	1·6% (0·4–4·0)	0·45	2·1% (0·7–4·8)	0·4% (<0·1–2·3)	0·22
Redness							
	Any	7·0% (4·9–9·5)	7·8% (4·9–11·9)	0·66	2·1% (0·7–4·8)	3·4% (1·5–6·5)	0·42
	Severe	1·9% (0·9–3·5)	2·0% (0·6–4·5)	0·99	1·3% (0·3–3·6)	0·4% (<0·1–2·3)	0·62
Swelling							
	Any	8·3% (6·1–11·0)	6·3% (3·6–10·0)	0·32	2·9% (1·2–5·9)	4·2% (2·0–7·6)	0·45
	Severe	1·7% (0·8–3·3)	2·0% (0·6–4·5)	0·78	1·7% (0·5–4·2)	0·4% (<0·1–2·3)	0·37
Hardness							
	Any	19·9% (16·6–23·6)	22·0% (17·0–27·5)	0·51	11·7% (7·9–16·4)	15·6% (11·2–20·8)	0·22
	Severe	2·1% (1·1–3·8)	1·2% (0·2–3·4)	0·57	2·1% (0·7–4·8)	0·4% (<0·1–2·3)	0·22
Bruising							
	Any	12·4% (10·0–15·5)	9·8% (6·4–14·1)	0·29	8·8% (5·5–13·1)	9·2% (5·9–13·7)	0·85
	Severe	0·8% (0·2–2·0)	0·8% (<0·1–2·8)	1·0	0·4% (<0·1–2·3)	0	1·0
Itching							
	Any	19·0% (15·7–22·6)	22·8% (17·7–28·4)	0·22	12·5% (8·6–17·4)	13·0% (9·0–18·0)	0·86
	Severe	1·9% (0·9–3·5)	0·4% (<0·1–2·2)	0·11	0	0·8% (0·1–3·0)	0·50
At least one local reaction							
	Any	38·1% (33·9–42·4)	47·8% (41·6–54·1)	0·010	23·8% (18·5–29·6)	28·2% (22·5–34·3)	0·27
	Severe	6·6% (4·6–9·1)	4·7% (2·5–8·1)	0·30	4·2% (2·0–7·5)	1·7% (0·5–4·2)	0·17
**Dominant arm local injection-site reactions**							
Tenderness							
	Any	19·5% (16·2–23·2)	22·0% (17·0–27·5)	0·43	··	··	··
	Severe	1·4% (0·1–2·5)	1·2% (0·2–3·4)	0·99	··	··	··
Redness							
	Any	4·5% (2·8–6·6)	6·3% (3·6–10·0)	0·28	··	··	··
	Severe	0·6% (0·1–1·7)	2·4% (0·9–5·0)	0·066	··	··	··
Swelling							
	Any	4·1% (2·5–6·1)	6·3% (3·6–10·0)	0·18	··	··	··
	Severe	0·8% (0·2–2·0)	2·0% (0·6–4·5)	0·17	··	··	··
Hardness							
	Any	14·1% (11·2–17·4)	18·0% (13·5–23·3)	0·16	··	··	··
	Severe	0·8% (0·2–2·0)	1·2% (0·2–3·4)	0·69	··	··	··
Bruising							
	Any	9·3% (6·9–12·1)	9·4% (6·1–13·7)	0·95	··	··	··
	Severe	0·4% (0·1–1·4)	0	1·0	··	··	··
Itching							
	Any	12·8% (10·0–16·0)	18·0% (13·5–23·3)	0·050	··	··	··
	Severe	1·2% (0·4–2·5)	1·2% (0·2–3·4)	1·0	··	··	··
At least one local reaction							
	Any	26·7% (22·9–30·7)	38·8% (32·8–45·1)	0·001	··	··	··
	Severe	3·7% (2·2–5·7)	5·1% (2·7–8·6)	0·35	··	··	··
**Systemic reactions**							
Weakness or tiredness							
	Any	35·8% (31·6–40·1)	34·9% (29·1–41·1)	0·81	27·5% (22·0–33·6)	24·0% (18·7–29·9)	0·38
	Severe	6·0% (4·1–8·4)	2·8% (1·1–5·6)	0·050	6·3% (3·5–10·1)	2·9% (1·2–6·0)	0·084
Headache							
	Any	35·2% (31·1–39·5)	32·6% (26·8–38·7)	0·47	30·0% (24·3–36·2)	24·4% (19·1–30·3)	0·17
	Severe	6·0% (4·1–8·4)	2·0% (0·6–4·5)	0·011	4·2% (2·0–7·5)	2·5% (0·9–5·4)	0·32
Fever							
	Any	4·3% (2·7–6·4)	1·6% (0·4–4·0)	0·057	3·8% (1·7–7·0)	3·8% (1·7–7·1)	0·99
	Severe	0·8% (0·2–2·0)	0	0·31	0·4% (<0·1–2·3)	0·8% (0·1–3·0)	0·62
Joint pain							
	Any	19·5% (16·2–23·2)	20·0% (15·3–25·4)	0·88	16·3% (11·8–21·5)	11·8% (8·0–16·5)	0·16
	Severe	2·7% (1·5–4·5)	1·2% (0·2–3·4)	0·20	2·1% (0·7–4·8)	1·3% (0·3–3·6)	0·72
Muscle pain							
	Any	17·8% (14·6–21·4)	21·2% (16·3–26·7)	0·26	18·3% (13·6–23·8)	11·3% (7·6–16·1)	0·032
	Severe	2·3% (1·2–4·0)	2·0% (0·6–4·5)	1·0	2·1% (0·7–4·8)	0·8% (0·1–3·0)	0·45
Rigors							
	Any	5·8% (3·9–8·2)	5·5% (3·0–9·0)	0·86	3·8% (1·7–7·0)	3·8% (1·7–7·1)	1·0
Increased sweating							
	Any	8·9% (6·6–11·7)	7·1% (4·2–10·9)	0·38	5·0% (2·6–8·6)	7·6% (4·5–11·7)	0·25
At least one systemic reaction							
	Any	50·3% (45·9–54·7)	52·9% (46·6–59·2)	0·49	42·9% (36·6–49·4)	34·5% (28·4–40·9)	0·058
	Severe	10·4% (7·9–13·4)	5·5% (3·0–9·0)	0·022	8·3% (5·2–12·6)	5·5% (2·9–9·2)	0·22

Data are incidence with 95% CI in parentheses. Any reaction means all cases, irrespective of intensity.

Four maternal deaths occurred during the study period (ie, until 24 weeks after delivery), one in the single-dose group, one in the double-dose group, and two in the two-single-doses group; none of these deaths were attibuted to the vaccine (figure; appendix p 6). 22 infant deaths occurred up to age 24 weeks, of which seven were in the single-dose group, 11 were in the double-dose group, and four were in the two-single-doses group (figure; appendix pp 8–9). None of the infant deaths were attributed to maternal vaccination. Furthermore, 21 fetal deaths occurred, 11 in women in the two-single-doses group (of which three occurred before the second vaccination visit), five in the single-dose group, and five in the double-dose group (appendix p 7). Four fetal deaths occurred within 14 days of maternal vaccination, one in the single-dose group and three in the two-single-doses group (two after the first dose and one after the second dose), and were consequently deemed possibly related to maternal vaccination. However, this association was only based on temporal associations to vaccination (adverse event within 14 days of vaccination), which was decided a priori. The three women (including one twin pregnancy) did not report any local or systemic reactions after vaccination that could indicate a causative effect of the vaccine (appendix p 7). We saw no differences in the proportion of maternal, infant, or fetal deaths, or infant birthweights between the single-dose group and the other two study groups (table 1).

## Discussion

In pregnant women living with HIV, the use of a double dose of inactivated influenza vaccine slightly improved immunogenicity compared with a single dose, but at 1 month after vaccination fewer than 66% of women in the double-dose group seroconverted to the most immunogenic vaccine strain (ie, A/H1N1pdm09), which was lower than the 92% seroconversion seen in our 2011 cohort of pregnant women without HIV who were given a standard single dose of inactivated influenza vaccine.^5^ A second standard inactivated influenza vaccine dose did not improve the immune response in the current cohort of women. These results are in accordance with previous studies in non-pregnant women and men with HIV who had higher haemagglutination-inhibition antibody responses after high dose of inactivated influenza vaccine than after a single standard dose, but the responses were still inferior compared with those in adults without HIV.^11^, ^12^ Furthermore, a second dose of vaccine did not consistently increase seroresponses in pregnant or non-pregnant individuals living with HIV.^11^, ^13^, ^14^, ^15^, ^18^

Safety and immunogenicity of adjuvanted pandemic A/H1N1 vaccines have not been assessed in pregnant women with HIV, but immune responses in men and non-pregnant women living with HIV were higher with adjuvanted pandemic vaccines than with unadjuvanted pandemic vaccines.^14^, ^17^ A systematic review assessing the immunogenicity of different influenza vaccine strategies among individuals living with HIV found that, for the A/H1N1 strains, booster doses of adjuvanted vaccines containing 7·5 μg of antigen and 60 μg of single-dose vaccine (high-dose inactivated influenza vaccine) strategies induced higher rates of seroconversion and seroprotection than did standard doses.^23^ The 60 μg single-dose vaccine also emerged as the most immunogenic formulation for influenza-B strains, although no single strategy was significantly more immunogenic than the standard dose for A/H3N2.^23^ To our knowledge, only one study to date has assessed immune response to two doses of a high dose (30 μg per dose) A/H1N1 monovalent vaccine in pregnant women living with HIV.^18^ In that report, the second high-dose injection only slightly improved the proportion of women with haemagglutination-inhibition antibody titres of 1/40 or higher (73% after the first dose and 80% after the second dose) and who seroconverted (66% after the first dose and 72% after the second dose).^18^

Although our cohort was heterogeneous, the participants had weak haemagglutination-inhibition antibody immune responses after vaccination with the inactivated influenza vaccine despite having generally normal CD4 cell counts and a high proportion being on antiretroviral therapy (ART). Possible reasons for this observation include residual immune dysregulation affecting both T-cell and B-cell quantities and function, immune activation, and immunosenescence.^24^, ^25^ Richardson and Weinberg^10^ showed that regulatory T cells have an important role in attenuating immune responses to inactivated influenza vaccine in pregnant women living with HIV, with high proportions of influenza-specific regulatory T cells being associated with both reduced haemagglutination-inhibition antibody titres and influenza-specific cell-mediated responses.

In addition to protecting the mothers against infection with influenza virus, another potential benefit of inactivated influenza vaccine during pregnancy is to prevent infection in the neonates.^5^ Although our study was not powered to detect vaccine efficacy in infants exposed to HIV the incidence of influenza was similar in the three study groups. In our previous randomised controlled trial^5^ infants born to mothers with HIV who had been given inactivated influenza vaccine had significantly higher haemagglutination-inhibition antibody titres than those born to mothers who had been given placebo, but titres were lower than in infants born to women without HIV who were given inactivated influenza vaccine. Compared with infants in the single-dose group, those in the double-dose group had increased haemagglutination-inhibition titres only for A/H1N1pdm09, but the titres and the proportion of infants with titres of 1/40 or higher against A/H1N1pdm09 were still lower than previously described for infants born to mothers without HIV who had been given a single dose of inactivated influenza vaccine (median geometric mean titre ranged from 41·8 to 93·3 and proportion with haemagglutination-inhibition titres of 1/40 or higher ranged from 60·0% to 81·1% for the three strains).^5^ In the current study, we only did haemagglutination-inhibition assays in blood samples collected from infants in the single-dose and double-dose groups. The decision to forego the comparison of haemagglutination-inhibition titres at birth between infants exposed to single-dose and two-single-doses of inactivated influenza vaccine was based on the lack of benefit of the second dose of inactivated influenza vaccine on maternal haemagglutination-inhibition titres. Furthermore, we assumed that 28 days from time of vaccination to birth was the ideal interval for efficient transplacental transfer of antibodies,^26^ and almost 20% of infants were born before this interval.

The three vaccination regimens were well tolerated among pregnant women, with fewer than 48% of participants having at least one solicited local injection-site reaction and fewer than 53% having at least one systemic reaction. Except for a higher incidence of local reactions in women in the double-dose group, the safety profile was similar in the double-dose and two-single-doses groups compared with the single-dose group. Four fetal deaths occurred within 14 days of receipt of inactivated influenza vaccine and were attributed as being possibly related to the vaccination only due to the temporal association because no reactogenicity was reported in these mothers after vaccination. The incidence of fetal deaths in the current study—ie, 26 per 1000 births, is similar to the incidence of stillbirths estimated in 2015 in Soweto with administrative databases (22·5 stillbirths per 1000 births).^27^ None of the infant deaths were attributed to maternal inactivated influenza vaccine vaccination.

Limitations of this report include that the study was done during the transition of prevention of mother-to-child HIV transmission management to option B+, such that some women with CD4 counts of 350 cells per μL or higher were still given zidovudine monotherapy during pregnancy. This fact raises the question of whether differential use of ART might influence antibody responses to vaccination. However, we have previously reported in a study of single-dose inactivated influenza vaccine in pregnant women before option B+, that plasma HIV viral load and use of ART at the time of vaccination were not associated with seroconversion 1 month after vaccination.^6^ Similarly, other studies in people with HIV before three-drug ART was available also showed that the CD4 count threshold below which antibody responses to inactivated influenza vaccine were compromised was 200 cells per μL.^7^, ^28^ Women in this category were treated with three-drug ART in the current study. Collectively, these observations support the hypothesis that the effect of ART on antibody responses to the vaccine depends on the extent to which they promote immune reconstitution and the use of three-drug ART in the most immune-compromised women in the current study was more likely to have homogenised antibody responses to vaccination. Other study limitations were that only one influenza season was covered and a single inactivated influenza vaccine formulation was assessed at a single study site. Also, only 37% of participants had CD4 counts of less than 350 cells per μL. Although this low proportion prevents us from generalising our findings to a population with severe immunosuppression, it accurately represents the current landscape of HIV infection globally, in which individuals with HIV are treated earlier than in the past. By contrast, since only 27% of the women were virologically suppressed, a more in-depth analysis is needed to better understand the association between HIV viral load and immune responses to inactivated influenza vaccine. Another limitation was that only 397 (76%) of 525 infants were included in the analysis of haemagglutination-inhibition antibody titres on or before age 7 days, although they were distributed equally across study groups. Furthermore, the observed immunogenicity in the single-dose group was lower than anticipated in the sample size calculation, which could have affected the power of the study.

Despite lower humoral responses to influenza vaccination in individuals living with HIV, inactivated influenza vaccine is efficacious, and vaccination of this vulnerable population should continue.^5^, ^29^ Nonetheless, the results for the current study show that doubling the dose of antigen or administration of a second dose of non-adjuvanted inactivated influenza vaccine did not increase the immunogenicity of the vaccine in women living with HIV to levels similar to those of women without HIV. These findings emphasise the need for continued efforts to find an improved influenza vaccine or vaccination strategy for this population to improve protection of their infants, such as exploring the use of high-dose inactivated influenza vaccine that is currently approved for older adults in pregnant women with HIV.^30^

## Data sharing

De-identified individual participant data that underlie the results reported in this Article (text, tables, figures, and appendices) will be shared upon request. The data dictionary and study protocol are also available. Data will be available from 9 months after publication. Researchers who wish to use the data to address any specific questions not directly addressed under the study objectives and that the data would lend itself to and who provide a methodologically sound proposal that has been approved by an independent review committee can request the data. Proposals should be directed to nunesm@rmpru.co.za or madhis@rmpru.co.za. To gain access, data requestors will need to sign a data access agreement and any transfer of data will be governed by the terms of the local Ethics Committee. Data sharing will be done on a collaborative basis, with the study principal investigator (or their nominee) being included in any further interrogation of the data. The data will be provided in the format in which it has been entered at Respiratory and Meningeal Pathogens Research Unit with the necessary data dictionary.

## References

[1] Phadke VK, Omer SB (2016). Maternal vaccination for the prevention of influenza: current status and hopes for the future. Expert Rev Vaccines.

[2] Zaman K, Roy E, Arifeen SE (2008). Effectiveness of maternal influenza immunization in mothers and infants. N Engl J Med.

[3] Tapia MD, Sow SO, Tamboura B (2016). Maternal immunisation with trivalent inactivated influenza vaccine for prevention of influenza in infants in Mali: a prospective, active-controlled, observer-blind, randomised phase 4 trial. Lancet Infect Dis.

[4] Steinhoff MC, Katz J, Englund JA (2017). Year-round influenza immunisation during pregnancy in Nepal: a phase 4, randomised, placebo-controlled trial. Lancet Infect Dis.

[5] Madhi SA, Cutland CL, Kuwanda L (2014). Influenza vaccination of pregnant women and protection of their infants. N Engl J Med.

[6] Nunes MC, Cutland CL, Dighero B (2015). Kinetics of hemagglutination-inhibiting antibodies following maternal influenza vaccination among mothers with and those without HIV infection and their infants. J Infect Dis.

[7] Zanetti AR, Amendola A, Besana S, Boschini A, Tanzi E (2002). Safety and immunogenicity of influenza vaccination in individuals infected with HIV. Vaccine.

[8] Kroon FP, Rimmelzwaan GF, Roos MT (1998). Restored humoral immune response to influenza vaccination in HIV-infected adults treated with highly active antiretroviral therapy. AIDS.

[9] Durando P, Fenoglio D, Boschini A (2008). Safety and immunogenicity of two influenza virus subunit vaccines, with or without MF59 adjuvant, administered to human immunodeficiency virus type 1-seropositive and -seronegative adults. Clin Vaccine Immunol.

[10] Richardson K, Weinberg A (2011). Reduced immunogenicity of influenza vaccines in HIV-infected compared with uninfected pregnant women is associated with regulatory T cells. AIDS.

[11] El Sahly HM, Davis C, Kotloff K (2012). Higher antigen content improves the immune response to 2009 H1N1 influenza vaccine in HIV-infected adults: a randomized clinical trial. J Infect Dis.

[12] McKittrick N, Frank I, Jacobson JM (2013). Improved immunogenicity with high-dose seasonal influenza vaccine in HIV-infected persons: a single-center, parallel, randomized trial. Ann Intern Med.

[13] Lagler H, Grabmeier-Pfistershammer K, Touzeau-Römer V (2012). Immunogenicity and tolerability after two doses of non-adjuvanted, whole-virion pandemic influenza A (H1N1) vaccine in HIV-infected individuals. PLoS One.

[14] Launay O, Desaint C, Durier C (2011). Safety and immunogenicity of a monovalent 2009 influenza A/H1N1v vaccine adjuvanted with AS03A or unadjuvanted in HIV-infected adults: a randomized, controlled trial. J Infect Dis.

[15] Cooper C, Thorne A, Klein M (2011). Immunogenicity is not improved by increased antigen dose or booster dosing of seasonal influenza vaccine in a randomized trial of HIV infected adults. PLoS One.

[16] Cooper C, Klein M, Walmsley S (2012). High-level immunogenicity is achieved vaccine with adjuvanted pandemic H1N1(2009) and improved with booster dosing in a randomized trial of HIV-infected adults. HIV Clin Trials.

[17] Soonawala D, Rimmelzwaan GF, Gelinck LB, Visser LG, Kroon FP (2011). Response to 2009 pandemic influenza A (H1N1) vaccine in HIV-infected patients and the influence of prior seasonal influenza vaccination. PLoS One.

[18] Abzug MJ, Nachman SA, Muresan P (2013). Safety and immunogenicity of 2009 pH1N1 vaccination in HIV-infected pregnant women. Clin Infect Dis.

[19] National Department of Health (2013). The 2012 national antenatal sentinel HIV and herpes simplex type-2 prevalence survey, South Africa.

[20] (2014). National Institute for Communicable Diseases monthly surveillance report. Report for 1 January to 31 January 2014. http://www.nicd.ac.za/assets/files/Monthly%20NICD%20Surveillance%20Report%20-%20January%202014.pdf.

[21] Weinberg A, Song LY, Walker R (2010). Anti-influenza serum and mucosal antibody responses after administration of live attenuated or inactivated influenza vaccines to HIV-infected children. J Acquir Immune Defic Syndr.

[22] Harris PA, Taylor R, Thielke R, Payne J, Gonzalez N, Conde JG (2009). Research electronic data capture (REDCap)—a metadata-driven methodology and workflow process for providing translational research informatics support. J Biomed Inform.

[23] Zhang W, Sun H, Atiquzzaman M, Sou J, Anis AH, Cooper C (2018). Influenza vaccination for HIV-positive people: systematic review and network meta-analysis. Vaccine.

[24] Effros RB, Fletcher CV, Gebo K (2008). Aging and infectious diseases: workshop on HIV infection and aging: what is known and future research directions. Clin Infect Dis.

[25] Lange CG, Lederman MM, Medvik K (2003). Nadir CD4+ T-cell count and numbers of CD28+ CD4+ T-cells predict functional responses to immunizations in chronic HIV-1 infection. AIDS.

[26] Blanchard-Rohner G, Meier S, Bel M (2013). Influenza vaccination given at least 2 weeks before delivery to pregnant women facilitates transmission of seroprotective influenza-specific antibodies to the newborn. Pediatr Infect Dis J.

[27] Madhi SA, Briner C, Maswime S (2019). Causes of stillbirths among women from South Africa: a prospective, observational study. Lancet Glob Health.

[28] Malaspina A, Moir S, Orsega SM (2005). Compromised B cell responses to influenza vaccination in HIV-infected individuals. J Infect Dis.

[29] Madhi SA, Maskew M, Koen A (2011). Trivalent inactivated influenza vaccine in African adults infected with human immunodeficient virus: double blind, randomized clinical trial of efficacy, immunogenicity, and safety. Clin Infect Dis.

[30] Grohskopf LA, Sokolow LZ, Broder KR, Walter EB, Fry AM, Jernigan DB (2018). Prevention and control of seasonal influenza with vaccines: recommendations of the Advisory Committee on Immunization Practices-United States, 2018–19 Influenza Season. MMWR Recomm Rep.

