# Supplementary material for: Immunogenicity and safety of different dosing schedules of trivalent inactivated influenza vaccine in pregnant women with HIV: a randomised controlled trial
Source: Lancet HIV. 2020 Jan 3;7(2):e91–e103. doi: 10.1016/S2352-3018(19)30322-4 (PMC7167514; doi:10.1016/S2352-3018(19)30322-4)
Supplement: Supplementary appendix [file mmc1.pdf]

THE LANCET
HIV

Supplementary appendix

This appendix formed part of the original submission and has been peer reviewed. We post it as supplied by the authors.

Supplement to: Nunes MC, Cutland CL, Moultri A, et al. Immunogenicity and safety of different dosing schedules of trivalent inactivated influenza vaccine in pregnant women with HIV: a randomised controlled trial. *Lancet HIV* 2020; published online Jan 3. http://dx.doi.org/10.1016/S2352-3018(19)30322-4.

Study inclusion criteria

Gestational age staging of the mother at enrolment was undertaken using a hierarchy of staging by fetal ultrasonagraphy (undertaken as part of standard of care), the last menstrual period of the mother and physical examination by palpation of fundal height.

(i) Pregnant women age ≥18 years to <39 years.

(ii) Gestational age ≥12 weeks to <36 weeks documented by the approximate date of the last menstrual period and corroborated by physical/ sonargraphic exam.

(iii) Documented to be HIV-infected on two assays prior to study-enrolment.

(iv) Able to understand and comply with planned study procedures.

(v) Provides written informed consent prior to initiation of study.

Study exclusion criteria

(i) Features of WHO clinical category 3 or 4 of AIDS at the time of enrolment.

(ii) Receipt of IIV, other than through the study, during the current influenza season documented by medical history or record.

(iii) Receipt of any live licensed vaccine ≤28 days or inactivated licensed vaccine (except tetanus toxoid) ≤14 days prior to study-vaccine.

(iv) Receipt of a non-licensed agent (vaccine, drug, biologic, device, blood product, or medication) ≤28 days prior to vaccination in this study or expects to receive another non-licensed agent before delivery unless study approval is obtained.

(v) Any significant (in the opinion of the site investigator) acute illness and/or oral temperature greater than or equal to 38C ≤24 hours prior to study entry.

(vi) Use of anti-cancer systemic chemotherapy or radiation therapy ≤48 weeks of study enrolment or has immunosuppression as a result of an underlying illness or treatment.

(vii) Long term use of glucocorticoids, including oral or parenteral prednisone ≥20 mg/day or equivalent for more than 2 consecutive weeks (or 2 weeks total) ≤12 weeks of study entry, or high-dose inhaled steroids (>800 mcg/day of beclomethasone dipropionate or equivalent) ≤12 weeks before study entry (nasal and topical steroids are allowed).

(viii) Receipt of corticosteroids for preterm labour ≤14 days before study entry.

(ix) Receipt of immunoglobulin or other blood products (with exception of Rho D immune globulin) ≤12 weeks prior to enrolment in this study or is scheduled to receive immunoglobulin or other blood products (with the exception of Rho D immune globulin) during pregnancy or for the first 24 weeks after delivery.

(x) Receipt of IL2, IFN, GMCSF or other immune mediators ≤12 weeks before enrolment.

(xi) Uncontrolled major psychiatric disorder.

(xii) History of a severe adverse reaction to previous IIV.

(xiii) Any condition that would, in the opinion of the site investigator, place the subject at an unacceptable risk of injury or render the subject unable to meet the requirements of the protocol.

(xiv) Pregnancy complications (in the current pregnancy) such as pre-term labour, hypertension (BP >140/90 in the presence of proteinuria or BP >150/100, with or without proteinuria or currently on antihypertensive medication) and pre-eclampsia.

Influenza virus testing

Investigation for influenza virus involved obtaining a nasopharyngeal aspirate from the infant and an oropharyngeal and nasal pharyngeal swabs using a flocked tipped plastic shaft swab (Tool and Carbide plastics (Pty) Ltd, South Africa) from the women. The samples were placed into viral transport medium and transferred to the RMPRU laboratory immediately following collection. Samples were than stored at -70C until testing was completed.

Randomisation process

Computer-generated randomisation lists, in blocks of 30: 10 in each arm (SD-IIV, DD-IIV and 2SD-IIV), were generated with assignment of consecutive 4-digit study numbers to the list. Block size of 30 was selected for practical and feasible reasons. Blocks were allocated consecutively to recruitment site when vaccine/placebo were requested. Once a block of 30 numbers had been allocated to a recruitment site, the envelopes for allocated block with randomisation forms were sent to recruitment site by pharmacist with the first set of vaccines prepared for the block. The randomisation forms, in envelopes with printed study number, were pre-printed with the 4-digit study number and an alphabetical and colour code for vaccine/placebo. Study medication was prepared by the pharmacist in syringes filled with either saline or with IIV as per randomisation.

Once an eligible participant had signed informed consent, the study doctor/nurse at recruitment site selected the next randomisation envelope (consecutive order) and selected a pair of syringes with alphabetical code which corresponds to the pre-printed code on the randomisation form. Vaccine/placebo were administered by the doctor/nurse in the deltoid muscle of each arm. Syringes containing IIV and placebo looked identical, however, for visit-1 vaccination; pharmacist ensured that the syringes were labelled ‘dominant’ and ‘non-dominant’. At enrolment, all participants received a dose of IIV in their non-dominant arm. Participants randomised to SD-IIV and 2SD-IIV groups received placebo in dominant arm, and participants in double-dose group received a second dose of IIV in the dominant arm. At visit-2, all participants received an injection in their non-dominant arm: SD-IIV and DD-IIV participants received placebo, and 2SD-IIV participants received IIV.

Study objectives

Primary objectives

1.1. Determine the seroresponse rate to each of the vaccine viral strains, defined as post-vaccination hemagglutination inhibition (HAI) levels of ≥1:40 and ≥4 fold increase over baseline HAI levels, in HIV-infected pregnant women receiving a double-strength IIV dose (DD-IIV) compared to mothers receiving a single-dose of IIV (SD-IIV) one-month after completion of the dosing schedule.

1.2. Determine the seroresponse rate to each of the vaccine viral strains, defined as post-vaccination HAI levels of ≥1:40 and ≥4 fold increase over baseline HAI levels, in HIV-infected pregnant women receiving two single-doses of IIV (2SD-IIV) spaced 28-35 days apart compared to women receiving SD-IIV one-month after completion of the dosing schedule.

1.3. Evaluate the safety of the three dosing schedules of IIV in HIV-infected pregnant women vaccinated between 12-36 weeks of gestational age.

Secondary objectives

2.1. Compare the proportion of newborns born to HIV-infected mothers in the DD-IIV and 2SD-IIV groups with HAI antibody titers of ≥1:40 to each of the three IIV strains to newborns of HIV-infected and HIV-uninfected women (enrolled in a separate cohort) who SD-IIV.

2.2. Determine the kinetics of transplacental transfer of maternal Hemagglutinin (HA) antibodies and persistence thereof until 24 weeks post-partum in the infants.

2.3. Compare the relative efficacy of a DD-IIV and 2SD-IIV schedule compared to a SD-IIV in pregnant HIV-infected women in protecting against laboratory-confirmed influenza illness in their infants up to 24 weeks of chronological age.

2.4. Compare the relative efficacy of a DD-IIV and 2SD-IIV schedule compared to SD-IIV schedule in pregnant HIV-infected women in protecting against protocol defined clinical influenza like illness (ILI) in their infants up to 24 weeks of chronological age.

2.5. Compare the relative efficacy of a DD-IIV and 2SD-IIV schedule compared to SD-IIV schedule in pregnant HIV-infected women in protecting against laboratory-confirmed influenza illness in maternal participants up to 24 weeks post-partum.

2.6. Compare the relative efficacy of a DD-IIV and 2SD-IIV schedule compared to SD-IIV schedule in pregnant HIV-infected women in protecting against protocol defined clinical ILI in maternal participants up to 24 weeks post-partum.

2.7. Define and compare cell mediated immune responses to different dosing options of IIV in HIV-infected pregnant women.

2.8. Evaluate the effect of IIV (SD-IIV, DD-IIV and 2SD-IIV) on CD4+ cell count and HIV-viral load changes comparing baseline levels to levels at delivery.

2.9. Describe safety outcome measures (maternal and foetal) of influenza vaccination of HIV-infected pregnant women.

2.10. Determine the impact of vaccination on T cell activation and regulatory B and T cells subpopulations pre-vaccination and one-month post-vaccination in HIV-infected pregnant women.

Table-S1. Immune responses to two single-doses of trivalent inactivated influenza vaccine in pregnant women living with HIV

	A/H1N1pdm09	A/H3N2	B/Yamagata
GMTs at baseline (95%CI)	9·5 (8·4, 10·8)	12·5 (11·0, 14·2)	5·9 (5·6, 6·2)
GMTs at 28-35 days post-first injection (95%CI)[a]	45·7 (38·0, 54·9)	45·7 (38·9, 53·6)	13·8 (12·2, 15·6)
GMTs at 28-35 days post-second injection (95%CI)	46·7 (39·7, 54·9)	44·1 (37·5, 51·9)	15·5 (13·9, 17·3)
p-value GMTs post-first vs. post-second injection[b]	0·68	0·51	0·008
HAI ≥1:40 at baseline; n (%)	31 (14·1)	51 (23·2)	2 (0·9)
HAI ≥1:40 at 28-35 days post-first injection; n (%)[a]	145 (65·9)	151 (68·6)	52 (23·6)
HAI ≥1:40 at 28-35 days post-second injection; n (%)	148 (67·3)	145 (65·9)	54 (24·6)
p-value HAI ≥1:40 post-first vs. post-second injection	0·76	0·54	0·82

GMTs: geometric means titers; 95%CI: 95% confidence interval; HAI: hemagglutination inhibition.
[a]significantly higher compared to baseline for all comparisons.
[b]p-value calculated by pair student-t test.

Table-S2. Maternal hospital admissions during the study-period not including deaths

	Single-dose[a]	Double-dose	Two Single-doses
Pregnancy related hospitalizations	24 (+2)	26	23
Delivery related hospitalizations	54 (+2)	46	55
Hospitalization for infections	10 (+3)	6	7
Haematological related hospitalizations	1	0	1
Neurological related hospitalizations	1	0	0
Cardiovascular related hospitalizations	1 (+1)	1	4
Chronic condition related hospitalizations	(+1)	1	4
Total number of maternal hospitalizations	91 (+3)	79	91
Total number of mothers hospitalized	83 (+9)	70	82

Only the primary diagnosis of each admission is included.

[a]In parenthesis number of women randomised to the two single-doses group with an event before second vaccine dose was given or who missed second vaccination visit.

Table-S3. Details of the maternal deaths

Days between first vaccination and death	Days between second vaccination and death	Days between vaccination and delivery	Study-group	Related to vaccination	Description	Category
81	Visit not done	Died while pregnant	Single-dose	No	On antiretroviral therapy, CD4+ cell count was 211 and HIV viral load <40 copies/ml at vaccination. Died following liver and multi-organ failure at 26 weeks gestational age.	Liver failure
176	146	131 after second dose	Two Single-doses	No	Recent started on antiretroviral therapy, CD4+ cell count was 37 and HIV viral load 695503 copies/ml at vaccination. Diagnosed with disseminated tuberculosis and started on treatment. Demised at home at 176 days post-vaccination.	Infection, disseminated tuberculosis
194	166	127 after second dose	Two Single-doses	No	Recent started on antiretroviral therapy, CD4+ cell count was 70 and HIV viral load 131055 copies/ml at vaccination. Diagnosed with Kaposi's sarcoma. Demised due to nosocomial sepsis.	Infection, AIDS
89	Visit not done	38	Double-dose	No	Not on antiretroviral therapy, CD4+ cell count was 366 and HIV viral load 235430 copies/ml at vaccination. Death at home, with one day history of acute febrile illness and diarrhoea.	Infection

Table-S4. Details of miscarriage or stillbirths

Days between first vaccination and death	Days between second vaccination and death	Gestational age (weeks)	Study-group	Related to vaccination[c]
13	Before visit	19	Single-dose	Possible[d]
28	Before visit	32	Single-dose	No
56	28	36	Single-dose	No
121	93	36	Single-dose	No
139	111	term	Single-dose	No
6	Before visit	17	Two Single-doses[a,b]	Possible[e]
6	Before visit	17	Two Single-doses[a,b]	Possible[e]
19	Before visit	27	Two Single-doses[a]	No
29	1	23	Two Single-doses	Possible[f]
53	24	30	Two Single-doses	No
87	59	31	Two Single-doses	No
92	64	term	Two Single-doses	No
102	74	term	Two Single-doses	No
143	115	term	Two Single-doses	No
153	125	35	Two Single-doses	No
155	127	term	Two Single-doses	No
51	23	32	Double-dose	No
57	29	term	Double-dose	No
66	38	term	Double-dose	No
89	62	35	Double-dose	No
109	81	30	Double-dose	No

[a]Mother miscarried before receiving second IIV dose.
[b]Twin pregnancy.
[c]Attribution based on temporal association of <14 days to vaccination and not necessarily causative.
[d]No local or systemic reactions following vaccination. Possible related to vaccination just on temporal association.
[e]Inevitable miscarriage. Mother had spontaneous cerebrovascular accident related to ischemia; hysterectomy due to retained placenta. Possible related to vaccination just on temporal association.
[f]Antepartum haemorrhage, no underlying risk factors. No local or systemic reactions following first and second vaccination. Possible related to vaccination just on temporal association.

Table-S5. Infant hospital admissions

	Single-dose[a]	Double-dose	Two Single-doses
Prematurity related hospitalizations	11 (+3)	12	9
Delivery/obstetric related hospitalizations	17	7	13
Congenital pneumonia	1	0	0
Meconium aspiration	0	0	1
Respiratory distress syndrome	11	4	7
Transient tachypnoea of the newborn	5	3	5
Hospitalization for infection	24	23	22
Neonatal sepsis	3	6	1
Respiratory	12	9	14
Nosocomial	0	0	1
Meningitis	2	1	1
Acute gastroenteritis	4	4	5
Conjunctivitis	0	2	0
Dermatological	2	1	0
Urinary tract	1	0	0
Congenital related hospitalizations	4	1	4
Dermatological related hospitalizations	0	0	1
Gastrointestinal related hospitalizations	0	1	1
Neurological related hospitalizations	1	1	0
Metabolic related hospitalizations	3	5	3
Total number of hospitalizations	60 (+3)	51	57
Total number of infants hospitalized	52 (+3)	42	49

Only the primary diagnosis of each admission is included.
[a]In parenthesis number of infants born to women randomised to the two single-doses group who were born before second vaccine dose was given.

Table-S6. Details of the infant deaths

Days between first vaccination and death	Days between second study product and death	Age at time of death (days)	Days between first vaccination and birth	Days between second study product and birth	Study-group	Related to vaccination	Description	Underlying Category
31	3	0	31	3	Single-dose	No[b]	Twin 1 born at 27 weeks gestational age (birth weight 900g) at home following spontaneous premature labour. Died within few minutes of birth in ambulance.	Complications of premature birth
120	84	0	120	84	Single-dose	No	Term baby. Delivery complicated by severe meconium aspiration and birth asphyxia	Obstetric related, meconium aspiration
32	4	1	31	3	Single-dose	No[b]	Second twin born by vaginal delivery at 27 weeks gestational age (birth weight 840 g). Admitted for supportive care and died on day-1 of life.	Complications of premature birth
58	30	1	57	29	Single-dose	No	Preterm birth (22 weeks gestational age), following unsuccessful tocolysis. Died one day later. Not ventilated.	Complications of premature birth
88	60	2	86	58	Single-dose	No	Preterm birth at 28 weeks gestation age (birth weight 755g). Only provided supportive care for respiratory distress and hypoglycaemia.	Complications of premature birth
96	68	19	77	49	Single-dose	No	19 day old, died at home following one day history of fever and difficulty with breathing. Suspected neonatal sepsis.	Infection
75		61	14	Before visit	Single-dose	No	Baby born at 36 weeks gestational age with multiple congenital abnormalities: holoprosencephaly, hydrocephaly, meningomyelocele, club feet. Died on day 61 of life at hospice.	Congenital abnormality
52	24	7	52	24	Two Single-doses	No	Term baby (birth weight 2,7 kg) born with severe birth asphyxia and meconium aspiration. Developed hypoxic ischemic encephalopathy. Deteriorated on mechanical ventilation and died on day 7 of life.	Obstetric related, meconium aspiration
99	71	51	48	20	Two Single-doses	No	Seven-week-old infant demised at home (in Zimbabwe) following 2-day history of fever.	Infection
158	130	84	74	46	Two Single-doses	No	Three-month-old infant diagnosed to be HIV-infected at 6 weeks of age. Had a 9 day history of cough, and also developed fever and difficulty with breathing. Demised at home, without being hospitalized.	Infection
191	156	166	25	Before visit	Two Single-doses[a]	No	Intentional poisoning.	Unnatural causes (not in hospital)
43	15	0	43	15	Double-dose	No	Twin born at 23 weeks gestational age (birth weight 505g), following spontaneous preterm labour and died within a few minutes of birth.	Complications of premature birth

35	2	0	35	2	Double-dose	No[b]	Twin 1 born at 23 week gestational age (birth weight 550g) following unsuccessful attempt at tocolysis. Died within a few minutes of birth.	Complications of premature birth
35	2	0	35	2	Double-dose	No[b]	Twin 2 born at 23 weeks gestational age (birth weight 515g), following unsuccessful attempt at tocolysis. Died within a few minutes of birth.	Complications of premature birth
45	17	2	45	17	Double-dose	No	Twin born at 23 weeks gestational age (birth weight 520g). Provided supportive care for hyaline membrane disease and died on day 2 of life.	Complications of premature birth
69	36	4	65	32	Double-dose	No	Multiple congenital abnormalities: prune belly syndrome, pubic diastasis and bladder exstrophy. Deteriorated post-surgical correction of bladder.	Congenital abnormality
142	113	7	135	106	Double-dose	No	Term baby with intrauterine growth retardation (birth weight 1500g). Developed necrotising enterocolitis and died on day 7 of life.	Infection, Sepsis
68	40	9	59	31	Double-dose	No	Twin 2 born at 35 weeks gestational age. Developed acute diarrhoea with severe dehydration and metabolic derangement. Also diagnosed with perforated necrotising enterocolitis. Died on day 9 of life following medical and surgical management.	Complications of premature birth
39	10	16	23	Before visit	Double-dose	No	16 day old infant died at home. No previous history of illness symptoms.	Sudden infant death syndrome
50	22	20	30	2	Double-dose	No	Died soon after arrival to hospital for suspected sepsis. Two day history of irritability	Infection, Sepsis
42	5	34	8	Before visit	Double-dose	No	Twin born at 30 weeks gestational age (birth weight 1515g). Hospitalised for respiratory distress syndrome and developed perforated necrotising enterocolitis requiring bowel resection. Developed extended-spectrum beta-lactamase -producing Klebsiella spp. and Candida parapsilosis sepsis. Deteriorated and died on day 35 of life.	Complications of premature birth
138	109	105	33	4	Double-dose	No	Three month old infant diagnosed to be HIV-infected, hospitalized for multilobar pneumonia. Also diagnosed with E. coli septicaemia and anaemia.	Infection, Respiratory

[a]Mother delivered before receiving second IIV dose.
[b]Attributed as not related following unbinding at end of the study, since event occurred after second injection, which was a placebo injection.

Immunogenicity and safety of different dosing schedules of trivalent influenza vaccine in HIV-infected pregnant women: a randomized controlled trial.

Sponsored by Gates Foundation (Grant number: OPP1002747)

Protocol Chair: Shabir A. Madhi

Protocol Primary Collaborators: Keith P Klugman (Bacteriology)
Adriana Weinberg (Immunology)

Protocol Scientific Collaborators: Kathleen Neuzil
Justin Oritz
Mark Steinhoff
Eric A. F. Simões

Principal Investigator: Clare Cutland

Clinical co-investigators:
Avye Violari (Paediatrician and HIV mother-to-child transmission coordinator)

Collaborator: Marietjie Venter (Respiratory Virus Unit, National Institute for Communicable diseases)
Nirvashni Dwarka (Obstetrician and Gynaecologist)

Protocol Chair
Shabir A. Madhi
University of Witwatersrand
Respiratory and Meningeal Pathogens Research Unit & DST/NRF Vaccine Preventable Diseases
Chris Hani Road, Chris Hani-Baragwanath Hospital; New Nurses Residence-11th Floor,
Bertsham 2013
Johannesburg
South Africa
Phone: +27 11 386 6137
FAX: +27 11 882 1872
E-mail: ShabirM@nicd.ac.za

Protocol Primary Collaborator
Keith P Klugman
William H Foege Professor of Global Health
The Rollins School of Public Health Emory University
1518 Clifton Road, N.E - Room 720
Atlanta, GA 30322 USA
Tel: + 1 404 712 9001
Fax: +1 404 727 590
E-mail: keith.klugman@emory.edu

Protocol Primary Collaborator
Adriana Weinberg
University of Colorado Denver
Mail Stop 8604
12700 E. 19th Avenue, Room 11126
Aurora, CO 80045
Phone: +1 303 724 4480
FAX: +1 303 724 4485
E-mail: adriana.weinberg@ucdenver.edu

Principal investigator
Clare Cutland
University of Witwatersrand
Respiratory and Meningeal Pathogens Research Unit & DST/NRF Vaccine Preventable Diseases
Chris Hani Road, Chris Hani-Baragwanath Hospital; New Nurses Residence-11th Floor,
Bertsham 2013, Johannesburg, South Africa
Phone: +27 11 983 4277
FAX: +27 86 674 0564
E-mail: cutlandc@rmpru.co.za

Study coordinators
Lee-Anne Stoltenkamp, Lydia Ledwaba
University of Witwatersrand
Respiratory and Meningeal Pathogens Research Unit & DS/NRF vaccine Preventable Diseases
Chris Hani Road, Chris Hani-Baragwanath Hospital; New Nurses Residence-11th Floor,
Bertsham 2013, Johannesburg
South Africa
Phone: +271 19382515
E-mail: stoltenkampl@rmpru.co.za; ledwabal@rmpru.co.za

Protocol Statistician
Locadiah Kuwanda
University of Witwatersrand
Respiratory and Meningeal Pathogens Research Unit & DST/NRF vaccine Preventable Diseases
Chris Hani Road, Chris Hani-Baragwanath Hospital; New Nurses Residence-11th Floor,
Bertsham 2013, Johannesburg
South Africa
Phone: +27 11 983 4283
E-mail: kuwandal@rmpru.co.za

Protocol Data Manager
Albert Maribe
University of Witwatersrand
Respiratory and Meningeal Pathogens
Research Unit & DST/NRF vaccine
Preventable Diseases
Chris Hani Road, Chris Hani-Baragwanath
Hospital; New Nurses Residence-11th Floor,
Bertsham 2013, Johannesburg
South Africa
Phone: +27 11 983 4283
E-mail: maribea@rmpru.co.za

Protocol Laboratory Scientist
Peter Adrian
University of Witwatersrand
Respiratory and Meningeal Pathogens
Research Unit & DST/NRF vaccine
Preventable Diseases
Chris Hani Road, Chris Hani-Baragwanath
Hospital; New Nurses Residence-11th Floor,
Bertsham 2013, Johannesburg
South Africa
Phone+27 11 983 4283
E-mail: adrianp@rmpru.co.za

Protocol Laboratory Scientist
Marta Nunes
University of Witwatersrand
Respiratory and Meningeal Pathogens
Research Unit & DST/NRF vaccine
Preventable Diseases
Chris Hani Road, Chris Hani-Baragwanath
Hospital; New Nurses Residence-11th Floor,
Bertsham 2013, Johannesburg
South Africa
Phone: +27 11 983 4262
E-mail: nunesm@rmpru.co.za

Protocol collaborator(Scientific/ virology)
Details: Marietjie Venter
Head, Respiratory Virus Unit, National
Influenza Centre
National Institute for Communicable diseases
Private bag x 4, Sandringham, 2131
South Africa
Tel: +27 11 555 0478
Fax:+27 11 386 6455
Cell: +27 82 902 0412
E-mail: marietjiev@nicd.ac.za

Protocol Laboratory Scientist
Florette K Treurnicht
Respiratory Virus Unit
National Institute for Communicable Diseases
E-mail: florettet@nicd.ac.za

Protocol Scientific Collaborators
Kathleen Neuzil
Director, Influenza Vaccine Project
PATH
Mail: PO Box 900922, Seattle, WA 98109
Street: 2201 Westlake Avenue, Suite 200,
Seattle, WA 98121
Phone: +1 206 285 3500
Fax: +1 206 285 6619
E-mail: kneuzil@path.org

Justin Ortiz
Influenza Vaccine Project
Mail: PO Box 900922, Seattle, WA 98109
Street: 2201 Westlake Avenue, Suite 200,
Seattle, WA 98121
Phone: +1 206 285 3500
Fax: +1 206 285 6619
E-mail: jortiz@path.org

Protocol collaborator
Nirvashni Dwarka, (Obstetrician)
Department of Obstetrics, Chris Hani Baragwanath hospital, Soweto, South Africa
Cell no. +27 82 325 1191
E-mail: ndwarka@mtnloaded.co.za

Protocol Scientific Collaborator
Eric A. F. Simões
Professor of Pediatrics,
Division of Infectious Diseases
The Children's Hospital
13123 East 16th Avenue
Aurora, CO 80045
Phone: + 1 720 777 6977
Fax: + 1 720 777 7295
E-mail: eric.simoes@ucdenver.edu

Protocol co-investigator
Avye Violari (Paediatrician and HIV mother-to-child transmission coordinator)
Perinatal HIV Research Unit,
Chris Hani Road, Chris Hani-Baragwanath Hospital; New Nurses Residence-12th Floor,
Soweto, Johannesburg
South Africa
Phone: 27 11 989 9707
E-mail: violari@mweb.co.za

Sub-investigators:
Anthonet Koen
Michelle Groome
Stephanie Jones
Lisa Jose
Niresha Govender
Andrea Hugo
University of Witwatersrand
Respiratory and Meningeal Pathogens Research Unit & DST/NRF vaccine Preventable Diseases
Chris Hani Road, Chris Hani-Baragwanath Hospital; New Nurses Residence-11th Floor,
Bertsham 2013, Johannesburg
South Africa
Phone: +27 11 983 4283

Table of contents

BACKGROUND ... 7
Influenza in HIV-infected individuals ... 8
Immunological responses to influenza vaccine in HIV-infected adults ... 10
Effectiveness of influenza vaccination in HIV-infected adults ... 13
Effect of pregnant-women TIV vaccination in mothers and young infants ... 14
Interaction between Streptococcus pneumoniae and influenza illness ... 16
Primary objectives ... 19
Secondary objectives ... 20
Other exploratory objectives ... 21
PROJECT FRAMEWORK ... 21
Study population ... 21
Selection and enrollment of participants ... 24
Inclusion Criteria ... 24
Exclusion Criteria ... 25
Concomitant Medication Guidelines ... 26
Disallowed/Precautionary Medications ... 26
Study description and assumptions ... 27
Participant enrolment ... 29
SAMPLE SIZE CONSIDERATIONS ... 31
INVESTIGATIONAL PRODUCT ... 32
Normal strength Study-vaccine formulation: ... **Error! Bookmark not defined.**
Preparation of Blinded Study Products ... 33
TIV ... 33
PLACEBO ... 33
Product Supply, Distribution and Pharmacy ... 33
Study Product Acquisition and Distribution ... 33
SUBJECT FOLLOW-UP: ... 34
Criteria for investigating for influenza illness in infants ... 35
Criteria for investigating for influenza in mothers ... 36
Influenza virus identification and molecular characterization ... 36
IMMUNOGENICITY STUDIES ... 38
Processing of MATERNAL and INFANT samples ... 39
HAI assays. ... 39
CMI assays ... 39
T-lymphocyte activation assays ... 40
HIV viral load, HIV-ELISA and CD4+ testing ... 40
Nasopharyngeal Streptococcus pneumoniae colonization studies: ... 40
Nasopharyngeal viral carriage studies: ... 40
Interaction between Streptococcus pneumoniae and influenza virus ... 40
PARTICIPANT MANAGEMENT ... 42
Toxicity Management ... 42
Participant Management ... 42
Criteria for Study Discontinuation ... 43
SAE Reporting Requirements for this Study ... 44
Grading Severity of Events ... 44
Expedited AE Reporting Period ... 44
Monitoring ... 44
Early stopping rules for safety ... 45
HUMAN SUBJECTS PROTECTION: INSTITUTIONAL REVIEW BOARD AND INFORMED CONSENT ... 47
PUBLICATION OF RESEARCH FINDINGS ... 47
BIOHAZARD CONTAINMENT ... 47

ANALYSIS 48
Safety 48
Immunology 49
Efficacy 50

Background

Influenza, a vaccine preventable disease, leads to 20% increase in hospitalizations and 30% increase in outpatient visit in young children (<3 years) in the USA [1]. Determining the contribution of influenza to early childhood morbidity and mortality in sub-Saharan Africa and potential to prevent influenza disease through vaccination may contribute to reducing childhood deaths; as vaccines are developed, licensed and available at reasonable cost. Infants under 6 months of age have the highest rate of influenza-attributable hospitalizations of any age group of children [1]. Unfortunately, although trivalent inactivated influenza vaccine (TIV) is safe in this age group it is poorly immunogenic and not licensed [2,3]. As pregnant women also have an increased risk of serious illness from influenza infection, one strategy to prevent the complications of influenza in pregnant women and young infants is through maternal TIV immunization. This could result in direct protection of the women and protection of the young infant consequent to transplacental transfer of TIV induced antibodies [4].

WHO recommends that pregnant women should receive TIV, however, adherence is poor in the few industrialized countries that have adopted the recommendation, and non-existent in low-middle income countries [5,6]. Barriers to administration of vaccines during pregnancy are abundant. This was studied with respect to seasonal influenza vaccines, which are recommended for all pregnant women in the USA and other well-resourced countries. Prior to the H1N1-2009 influenza pandemic (H1N1-2009pdm), only 10% of pregnant women received seasonal influenza vaccine in developed countries [7]. High levels of provider knowledge about maternal vaccination and positive attitudes towards influenza vaccination were identified as key elements favoring vaccination [8]. In contrast, the lack of information on effectiveness was a common deterrent for providers, whereas concerns about safety were invoked both by mothers and providers [5,7]. These same factors probably explain the virtual non-existent use of TIV in pregnant women from low-middle income countries, including South Africa. Only recently have data become available from another low-income country (Bangladesh), with a very low prevalence of HIV, in which the benefit of maternal TIV vaccination was investigated as a secondary outcome measure [9].

The limited data on the immunogenicity and efficacy of TIV in HIV-infected individuals in general, is a further impediment to the routine adoption of TIV immunization of pregnant

women as part of standard of care in sub-Saharan African countries. The latter is pertinent in Southern-African countries where the prevalence of HIV in pregnant women ranged between 15 and 39% in 2007 [10].

Influenza in HIV-infected individuals

There is a paucity of data regarding the burden of influenza virus in developing countries, including those with a high prevalence of HIV. There is also limited data on the epidemiology of influenza virus infection in HIV-infected individuals globally. A temporal association between an increase in the number of deaths during the influenza season in USA cities with a high prevalence of HIV infection, coupled with case reports of severe influenza-related morbidity in HIV-infected individuals was the impetus for recommending annual influenza vaccination by the Advisory Committee on Immunization Practices in the USA [11]. Subsequently, a study in the USA reported that influenza-attributable hospitalization rate in HIV-infected adults was 48 (95% confidence interval [CI]: 16-91) per 1000 persons in 1995 and decreased to 5 (95% CI: -0·5-11) per 1000 persons per year during 1996 through 1999, after the introduction of highly active antiretroviral treatment (HAART). Although influenza-associated hospitalizations had declined in patients with HIV infection in the post-HAART era, the rates remained comparable to rates in other high-risk groups for which annual influenza vaccination was recommended [12].

Much of the differences in morbidity and mortality in HIV-infected individuals with influenza-associated pneumonia may be due to contribution of other co-existing pathogens. Although there are case reports of severe influenza illness in HIV-infected adults, in general the spectrum and severity of influenza associated illness have been described to be similar between HIV-infected and HIV-uninfected adults [13-15]. There is however a trend for HIV-infected adults being more likely to be hospitalized, evaluated in an emergency room or have an illness of ≥14 days than HIV-uninfected adults (21% vs. 0%; P=0.06) [15]. There also does not appear to be any evidence for clinical, immunological or virological progression of HIV during the course of an influenza season [16].

In South Africa, the burden of influenza associated pneumonia has been characterized in HIV-infected children. The incidence of severe pneumonia in which influenza virus

was identified was 8.03 fold (95%CI: 5.05-12.76) greater in HIV-infected compared to HIV-uninfected children aged <2 years [17]. Although the duration of hospitalization for influenza associated pneumonia was similar between HIV-infected and HIV-uninfected children (median 4-5 days), there was a statistically non-significant increased risk of mortality among HIV-infected children (8.0% vs. 2.2%; P=0.20) [18]. Furthermore, differences in the clinical spectrum of influenza-associated pneumonia include HIV-infected children remaining at risk of severe influenza pneumonia beyond early infancy, which is the major age-risk group in HIV-uninfected children, as well as there being more severe chest radiographic changes in HIV-infected children. The latter may be related to impairment of cell mediated immunity, which is key to controlling influenza virus replication and which is compromised in HIV-infected individuals.

Although there are no specific data on the burden of influenza illness in HIV-exposed-uninfected infants, these infants are increasingly being recognized as having greater morbidity and mortality than infants born to HIV-uninfected mothers. The biological reason for this is yet to be fully characterized, although aberrations of the immune system of HIV-exposed-uninfected children have been described [19-21]. This may relate to exposure to HIV virions in-utero, which may affect responses to HIV-unrelated antigens. Differences in immune responses to BCG vaccine in HIV-exposed-uninfected children were found by van Rie et al. [22], however these findings were not confirmed in a more recent study [23]. Additionally, Mansoor et al. did not show any difference in naive, memory, effector and senescent T-cell subsets during the first year of life between HIV-uninfected infants born to HIV-infected and HIV-uninfected mothers [24]. The results from a study on 7-valent pneumococcal conjugate vaccine (PCV7) failed to find any strong association in antibody responses to the vaccine between HIV-unexposed-uninfected and HIV-exposed-uninfected infants [25]. This suggested that the heightened morbidity and mortality experienced by HIV-exposed-uninfected children was unlikely due to immunological aberrations. Another possibility for the increased morbidity and mortality, primarily experienced during early infancy in HIV-exposed-uninfected children may relate to impaired transplacental antibody transfer compared to newborns of HIV-uninfected mothers. This may consequently affect any strategy targeting vaccination of mothers in relation to optimizing protection against the targeted pathogen in the mother as well as to their newborn in women who are HIV-infected.

Immunological responses to influenza vaccine in HIV-infected adults

Similar to high-risk HIV-uninfected individuals, the mainstay for the prevention of influenza virus infections in HIV-infected individuals is immunization with the trivalent sub-unit vaccines. Initial concerns regarding the use of the sub-unit vaccines, which elicit a T-cell dependent immune response, centered around the transient increase in HIV viral load and decreases in CD4+ lymphocyte counts that was observed post-vaccination in HIV-infected individuals [26]. These changes however are infrequent (4-18%), resolved at later time-points and are considered to be clinically non-significant [27-29]. The frequency of transient increases in HIV viral load is less common in HIV-infected individuals when vaccinated whilst stable on HAART as well as in HIV-infected adults with CD4+ cell counts of 200-500 cells/μl at the time of vaccination [28,30]. The changes in viral load, even in HIV-infected individuals on HAART and virologically suppressed (viral load <400 copies/ml), are thought to be due to in vivo mobilization of latently infected cell reservoirs [31].

In general, immune responses to the sub-unit influenza vaccine are lower in HIV-infected individuals. The immunogenicity to influenza vaccination seems to correlate directly with CD4+ cell count and inversely with HIV viral load [32,33]. In particular, antibody responses are impaired in HIV-infected adults with CD4+ cell counts of <100/μl and HIV RNA levels of >100 000 copies/ml [32]. In HIV-infected adults, protective concentration of antibodies have been observed in 13-50% of subjects with AIDS and in 52-89% of asymptomatic HIV-infected adults, compared to 94-100% of HIV-uninfected adults [34]. The preliminary results from a trial conducted in Soweto in 2011 support these results.

In 2011, pregnant women were enrolled onto two Maternal influenza vaccination trials being conducted in the Soweto community, one in HIV-infected women, and the other in HIV-uninfected women. In both these trials (HREC reference number 101106 and 101107) the immunogenicity of a single dose of TIV administered between 20 and 36 weeks of gestation was investigated.

HIV-uninfected women showed a good response one month post-vaccination with 85.3%, 92.7% and 98.5% of women having sero-protective levels of antibodies to the vaccine strains, H3N2, H1N1 and B respectively, however, HIV-infected women showed an inferior response to vaccination (see table 1). The CD4+ cell count at baseline (prior

to vaccination) affected the immunological response mounted by HIV-infected women: 12.5%, 12.5% and 62.5% of women with CD4+ cell count <200, and 50%, 66.7% and 77.8% of women with CD4+ count ≥500 had sero-protective levels to vaccine strains, H3N2, H1N1 and B respectively, one month post-receipt of single-dose TIV.

Table 1: Comparison of proportion of HIV-uninfected and HIV-infected pregnant participants with sero-protective antibody levels against vaccine-type influenza strains (2011) one month post receipt of single-dose TIV

	HIV-uninfected	HIV-infected women, stratified by CD4+ cell results at baseline								
	n=68	CD4+<200 n=8	CD4+≥200 n=65	p-value	CD4+<350 n=30	CD4+≥350 n=43	p-value	CD4+<500 n=55	CD4+≥500 n=18	p-value
H3N2 % (n)	85.3 (58)	12.5 (1)	50.8 (33)	0.06	33.3 (10)	55.8 (24)	0.31	45.5 (25)	50.0 (9)	0.06
H1N1 % (n)	92.7 (63)	12.5 (1)	73.9 (48)	0.001	53.3 (16)	76.7 (33)	0.036	67.3 (37)	66.7 (12)	0.96
B % (n)	98.5 (67)	62.5 (5)	70.8 (46)	0.63	63.3 (19)	74.4 (32)	0.31	67.3 (37)	77.8 (14)	0.56

Influenza vaccination of HIV-infected adults is also less likely to be associated with the generation of influenza specific memory B cells than in HIV-uninfected adults [35].

It has been shown that two-doses of an inactivated Influenza A (H1N1) 2009 monovalent vaccine given 21-28 days apart to HIV-infected pregnant women was safe and immunogenic and that this regimen may achieve higher rates of antibody sero-protection than a standard single vaccine dose [36].

Effectiveness of influenza vaccination in HIV-infected adults

In keeping with the modest immune responses observed to the sub-unit vaccines, a recent meta-analysis confirmed that influenza vaccines are moderately effective in reducing the incidence of influenza in HIV-infected individuals. The meta-analysis included the only four published studies evaluating the effectiveness of influenza vaccination in HIV-infected individuals [37]. Two of these studies were performed in highly-selected groups in the USA. One among 102 HIV-infected military recruits [38] and another observational study among 71 HIV-infected adults in a residential facility [15]. The other two studies, both of which were non-randomized and published in 2005, were performed among 145 HIV-infected adults in Italy [39] and another among 328 HIV-infected adults in Japan [40]. The vaccine efficacy in HIV-infected adults in the studies reviewed ranged between 27-78%, with the lowest vaccine effectiveness (27%) being observed in the study by Fine et al. [15], in which participants had a lower median CD4+ cell count (149 cells/μl) compared with 403 cells/μl in the study among USA Military recruits (vaccine efficacy 41%) and 379 cells/μl in the study from Japan (vaccine effectiveness 71%).

The incidence of “influenza illness”, the outcome-measure which differed between studies in terms of diagnosis as well as in relation to severity, ranged between 21% in the Japanese study to 62% in the study by Fine et al. and in Italy [15,39,40]. The incidence of disease in the only placebo-controlled, randomized study by Tasker et al. [38], which used an outcome of either respiratory symptoms with at least a four-fold increase in antibody titer and/or a positive viral culture, was 49% among unvaccinated and 29% in vaccinated individuals. This study too had limitations including relatively passive follow-up for influenza illness. Although impossible to make direct comparisons between these studies because of differences in case-ascertainment and outcome diagnosis, the high

incidence among unvaccinated individuals in all the studies indicate that influenza probably contributes to significant morbidity in HIV-infected adults.

The immunogenicity and efficacy of TIV in HIV-infected adults was only recently documented in an African setting. This placebo-controlled trial was approved by the University of the Witwatersrand Ethics Committee in 2008. This community-based randomized, placebo controlled trial reported that TIV was associated with a 75% reduction in influenza-confirmed illness [41]. The results of the study also confirmed the safety of TIV among African HIV-infected adults. The study, however, only included 7 women who were pregnant. In addition to no differences in solicited adverse event rates, there was also no difference in either CD4+ cell count changes or HIV viral load in those on antiretroviral treatment between TIV vaccinees compared to placebo recipients. This allayed previous concerns regarding the potential negative effect of TIV which centered around the observed transient increase in HIV viral load, even in HIV-infected individuals on HAART and who were virologically suppressed (viral load <400 copies/ml) [26,31]. Decreases in CD4+ cell counts have also been observed in HIV-infected individuals post-TIV vaccination [26]. These changes, however, even in past studies were infrequent (4-18%) and were considered to be clinically non-significant [27-29]. Preliminary data from 2011 Maternal influenza trials in HIV-infected and HIV-uninfected pregnant women supports the safety data described above: there were no significant differences in local or systemic adverse events reported by HIV-infected compared to HIV-uninfected pregnant women, and TIV did not have a significant impact on CD4+ cell and viral loads in HIV-infected participants.

Effect of pregnant-women TIV vaccination in mothers and young infants

The immunogenicity of TIV in pregnant women is similar to non-pregnant women, and high transplacental transfer of maternal influenza specific IgG antibody occurs (87 to 99%) to newborns; of which antibody half-life is 43-53 days [42,43]. Whilst a 65% reduction in acute respiratory illness in infants born to TIV-vaccinated (10.9%) vs. –unvaccinated (31%) mothers has been documented in the USA [44], two other observational studies failed to demonstrate a benefit of maternal immunization on neonatal acute respiratory illness [45,46]. A crucial prospective controlled study in Bangladesh, however, showed a 63% (95%CI: 5 to 85) reduction in laboratory-confirmed influenza illness in infants under 24 weeks of age born to mothers vaccinated with TIV [9]. The Bangladeshi study also

documented a 36% reduction in clinical illness in vaccinated mothers [9]. This compared favorably to a meta-analysis on effectiveness of TIV in non-pregnant healthy adults in which a 67% reduction in influenza confirmed illness and 23% reduction of clinically diagnosed illness were documented [47]. There has, however, not been any study on the effectiveness of maternal immunization with TIV on influenza- associated morbidity and mortality either in the mothers or infants in African settings.

Despite the encouraging results on maternal immunization from Bangladesh, and the data supporting that TIV is efficacious mainly in HIV-infected non-pregnant adults, preliminary results from the Maternal influenza trial being conducted in Soweto in 2011 highlight the reduced immune response mounted by HIV-infected, pregnant women to TIV. Further data on immune response to single-, double-, and two-doses of TIV are needed to advocate for routine use of, and finalize dosing schedule of TIV during pregnancy in settings with a high prevalence of HIV. HIV infection is known to decrease placental integrity and lower antibody levels in the fetus and newborn [48]. Furthermore, maternal hypergamma-globulinemia that is characteristic of HIV-infection may be associated with decreased neonatal antibody levels. This is supported by a study on the kinetics of measles antibody transfer during pregnancy in which although African women had higher total and measles-specific IgG levels, German women transferred a higher concentration of IgG to their infants [49]. This paradox is explained by the limited number of placental antibody receptors, resulting in IgG antibodies competing for available receptors and thereby decreasing vaccine-specific antibody transport [50]. In addition to the contribution of HIV, elevated IgG levels in African HIV-uninfected women may also be related to higher exposure to other pathogens as postulated in the above study [49]. Finally, because preterm birth increase with HIV infection, chronic maternal disease or malnutrition, transfer of maternal antibodies which is gestational age dependent, may be more affected by maternal immunization in sub-Saharan Africa where these processes are common [50]. The need to corroborate the findings from studies in other population groups is supported by the potential differences in immunogenicity of vaccines. In addition, more importantly in the context of this proposal, are the potential differences which may exists between populations with regard to transplacental antibody transfer during pregnancy to confer “passive protection” during early infancy.

The overall aim of this project is to evaluate the safety and immunogenicity of 3 different dosing options of TIV vaccination of HIV-infected pregnant women as follows:

1. Single dose (15µgHA/strain) TIV (SD-TIV),
2. Double dose (30µgHA/strain) TIV on same day (DD-TIV)
3. Two single (15µgHA/strain) doses of TIV one month apart (2SD-TIV)

Interaction between Streptococcus pneumoniae and influenza illness

Much of influenza virus associated morbidity and mortality may be due to the synergistic lethality of influenza with bacterial pathogens leading to pneumonia. Superimposed bacterial infections, especially *Streptococcus pneumoniae* and in patients treated with antibiotics *Staphylococcus aureus*, contribute to a large proportion (28-65%) of pneumonia deaths associated with influenza illness during pandemics [51,52]. The interaction of influenza virus predisposing to bacterial infection is also supported by other ecological studies and animal-model experiments [53-57]. Also increasingly recognized in the USA where in 2009 14 (37.8%) of the 37 influenza-associated childhood deaths bacterial co-infection was confirmed by bacterial culture from normally sterile sites [58]. Additionally, bacterial co-infections primarily due to *Streptococcus pneumoniae* were documented in 43% of childhood deaths related to H1N1-2009pdm influenza strain in the USA; and in 29% of autopsies from all ages with non-PCV vaccine types predominant due to the widespread direct and indirect effect of reducing the risk of pneumococcal infections since the introduction of PCV into the USA in 2000 [59,60].

The lack of sensitive diagnostic assays for diagnosing bacterial pneumonia has meant that vaccine trials are an essential way to investigate the role of bacteria in influenza –related morbidity and mortality. A trial of PCV published in Nature Medicine, conducted in this community in Soweto showed that PCV recipients had 45% less influenza –related hospitalization for pneumonia [61]. Pneumococcal colonization is an essential prelude to pneumococcal infection and the prevention of pneumococcal colonization as a surrogate for protection against pneumonia is the subject of a Gates Foundation Grand Challenge grant (PneumoCarr Project) in which this site is a key-partner. PCV given to young infants according to the WHO recommended schedule at 6, 10, and 14 weeks did not prevent existing pneumococcal colonization at 18 weeks of age (126 days) in Soweto, but rather prevented acquisition of new colonization which is apparent by 9

months of age (270 days) [62]. Influenza may thus facilitate the early acquisition of pneumococcal colonization and disease, and TIV vaccination of pregnant mothers may prevent this early acquisition. Early acquisition of pneumococcal carriage and infection may explain the significant impact of TIV in Bangladesh compared to the lack of effectiveness in preventing early infant sepsis from some USA studies, as pneumococcal colonization occurs early in life in developing countries and is associated with exacerbation of influenza infection [61,63].

Unpublished data, kindly shared by Dr Saad Omar (personal communication) from the Bangladeshi demonstration study, on which this grant funding is based, show that Bangladeshi infants of mothers who received TIV (with PCV7 given to infants) were better protected from acquisition of pneumococcal carriage during influenza season than infants of TIV-unimmunized mothers. No data in the African setting are available to support or refute this observation from Bangladesh. Introduction of PCV in low-income countries is a priority of GAVI, with 7 African countries already approved, and many others to follow, to introduce PCV within the next five years [61].

Breast-feeding

Essential nutritive and immunological components present in breast-milk makes it the first choice for infant nutrition. Currently, most national and international authorities, including the World Health Organization and United Nations Children's Fund, recommend 6 months of exclusive breastfeeding.

Fully breastfed infants have been shown to have lower overall illness rates, whereas minimal breastfeeding has not been found to be protective. (75) Breastfeeding duration also affects child morbidity. A study in the USA found an increased risk of respiratory tract infection including pneumonia and recurrent Otitis Media in children who were fully breastfed for 4 months vs. 6 months (76).

Regarding influenza infection, previous studies in animal models have shown that breast-feeding may decrease susceptibility to influenza as a result of passive antibody transfer. (77) Whereas it is appreciated that immunity can be transferred from mother to infant through breast-milk following maternal influenza exposure, the benefits granted by

breastfeeding as such are still not well explored. Exclusive breast-feeding is being encouraged in HIV-infected women.

This proposal that specifically targets HIV-infected women and their infants, will evaluate the safety and immunogenicity of three different dosing schedules of maternal TIV vaccination in HIV-infected women and their infants. Separate studies in which HIV-uninfected women are randomized to receive either single dose TIV or placebo from the same community were initiated in 2011 and will continue into 2013, and will be used as comparator groups.

Study objectives

The randomized trial will evaluate the safety and immunogenicity of three different dosing options of TIV (single-dose (SD-TIV), double-dose (DD-TIV), and two single-doses (2SD-TIV)) in HIV-infected pregnant women; and determine the dynamics of transplacental anti-influenza antibody transfer to their newborns and the kinetics thereof during early infancy. Although this study will only enroll HIV-infected women, a parallel cohort of HIV-uninfected women and their newborns will be enrolled from the same population in whom the safety, immunogenicity and efficacy of a single-dose of TIV is to be studied. The immunogenicity subgroup within the HIV-uninfected cohort will be used as a comparator group for one arm of our study population of HIV-infected pregnant women.

Primary objectives

1.1. Determine the sero-response rate to each of the vaccine viral strains, defined as post-vaccination hemagglutination inhibition (HAI) levels of ≥1:40 AND ≥4 fold increase over baseline HAI levels, in HIV-infected pregnant women receiving a double-strength TIV dose (DD-TIV) compared to mothers receiving a single-dose of TIV (SD-TIV) one-month after completion of the dosing schedule.

1.2. Determine the sero-response rate to each of the vaccine viral strains, defined as post-vaccination HAI levels of ≥1:40 AND ≥4 fold increase over baseline HAI levels, in HIV-infected pregnant women receiving two single-doses of TIV (2SD-TIV) spaced 28-35 days apart compared to women receiving a single-dose of TIV one-month after completion of the dosing schedule.

1.3. Evaluate the safety of the three dosing schedules of TIV in HIV-infected pregnant women vaccinated between 12-36 weeks of gestational age.

Secondary objectives

2.1. Compare the proportion of newborns born to HIV-infected mothers in the DD-TIV and 2SD-TIV arms with HAI antibody titers of ≥1:40 to each of the three TIV strains to newborns of HIV-infected and HIV-uninfected women (enrolled in a separate cohort) who received single-dose of TIV (SD-TIV).

2.2. Determine the kinetics of transplacental transfer of maternal Hemagglutinin (HA) antibodies and persistence thereof until 24 weeks post-partum in the infants.

2.3. Compare the relative efficacy of a DD-TIV and 2SD-TIV schedule compared to a single-dose TIV schedule in pregnant HIV-infected women in protecting against laboratory-confirmed influenza illness in their infants up to 24 weeks of chronological age.

2.4. Compare the relative efficacy of a DD-TIV and 2SD-TIV schedule compared to a single-dose TIV schedule in pregnant HIV-infected women in protecting against protocol defined clinical influenza like illness (ILI) in their infants up to 24 weeks of chronological age.

2.5. Compare the relative efficacy of a DD-TIV and 2SD-TIV schedule compared to a single-dose TIV schedule in pregnant HIV-infected women in protecting against laboratory-confirmed influenza illness in maternal participants up to 24 weeks post-partum.

2.6. Compare the relative efficacy of a DD-TIV and 2SD-TIV schedule compared to a single-dose TIV schedule in pregnant HIV-infected women in protecting against protocol defined clinical ILI in maternal participants up to 24 weeks post-partum.

2.7. Define and compare cell mediated immune responses to different dosing options of TIV in HIV-infected pregnant women.

2.8. Evaluate the effect of TIV (SD-TIV, DD-TIV and 2SD-TIV) on CD4+ cell count and HIV-viral load changes comparing baseline levels to levels at delivery.

2.9. Describe safety outcome measures (maternal and foetal) of TIV-vaccination of HIV-infected pregnant women.

2.10. Determine the impact of vaccination on T cell activation and regulatory B and T cells subpopulations pre vaccination and one month post vaccination in HIV-infected pregnant women.

Other exploratory objectives

- Determine the safety of TIV in HIV-infected women against poor obstetric outcomes including: low birth weight (<2 500 g), premature delivery (<37 weeks), emergency caesarean section and early-onset (<3 days) neonatal sepsis.
- Compare the proportion of newborns born to HIV-infected mothers in the DD-TIV and 2SD-TIV arms with HAI antibody titers of ≥1:80, ≥1:160, ≥1:320 and ≥1:640 to each of the three TIV strains to newborns of HIV-infected and HIV-uninfected women (enrolled in a separate cohort) who received single-dose of TIV.
- Explore vaccine efficacy of the 3 different dosing schedules in the infants stratified and/or adjusting for breast-feeding practices.
- Assess the association between breast-milk influenza IgA antibodies and serum IgG antibody concentrations, and the association thereof with protection against influenza confirmed illness in the infant.
- Define and compare cell mediated immune responses and T cell activation responses in infants born to HIV-infected women vaccinated with different dosing options of TIV.
- Define cell mediated immune responses to natural and breakthrough influenza infections 24 weeks after vaccination.

Project Framework

Study population

General site description

The study will be undertaken in Soweto (i.e. District D of Johannesburg, South Africa), an urban low-income community of 1.4 million Black-African inhabitants of a diversity of

Ethnic backgrounds, including Zulu, Xhosa and Sotho heritage. Although the majority of households have access to running water, 25% of families live in informal settlements and use fossil fuels for heating and cooking. The community is severely affected by HIV and the under-5 mortality rate in South Africa, including the study site, increased from 60 to 69 per 1 000 live births between 1990 and 2005 [64].Twelve percent of all deaths occur in the neonatal period and the infant mortality rate is 38 per 1 000 live births [65].

Health care facilities:

There are 23 primary health care (PHC) clinics in the Soweto region and a single public hospital; i.e. Chris Hani-Baragwanath Hospital (CHBH) which is the sole referral hospital for all the PHC clinics. Ninety-nine percent of women will attend antenatal visit prior to delivery at one of seven PHC antenatal clinics or at CHBH. Each year there are 28 500 births in the community, including approximately 21 000 which occur at CHBH and the others at PHC antenatal clinics. The largest of the antenatal clinics is at Lillian Ngoyi clinic, which neighbors CHBH. CHBH primarily provides secondary and tertiary level care to the population of Soweto, with approximately 90% of all hospitalizations from the community occurring here. All health care for pregnant women and children <6 years, including diagnostic tests and treatment, provided at the hospital and PHC clinics are free. Although there is a wide network of private general practitioners and traditional healers to where health care may be sought, CHBH is the primary source of referral from these health workers for serious illnesses. The immunization coverage rates for DTP3 and Hib conjugate vaccine (HibCV) ranged between 85%-90% in districts in the Soweto region in 2008 (Source: Department of Health Immunization Program and Gauteng Health).

HIV testing and HIV management:

All HIV testing is undertaken by trained counselors with pre- and post-test counseling provided to mothers either as part of standard of care. The prevalence of HIV infection in women attending antenatal clinics in Soweto has stabilized at 30% since 2005, and the vertical transmission of HIV has declined to 3% since 2008 (personal correspondence: Dr A Violari- head of prevention mother-to-child transmission program [PMTCT] program). HIV-infected mothers and children are provided with free HAART through established HIV clinics at CHBH in accordance with national guidelines for treatment. In addition to HAART where indicated, pregnant HIV-infected women are provided with

zidovudine (300mg BD) from 14 weeks of gestational age and a single dose of nevirapine (200mg), three-hourly zidovudine (300mg) during labor and single dose (500mg) Truvada post-partum and newborns are provided with nevirapine within 72 hour of birth and one week of zidovudine. Ninety-five percent of mothers agree to HIV tests during pregnancy. Currently, CD4+ cell count evaluation is undertaken on all mothers diagnosed as being HIV seropositive. Triple drug antiretroviral therapy is initiated in those mothers in whom the CD4+ cell count is <350 cells/μl. The coverage of HAART of pregnant women requiring triple therapy is estimated to be 75% at the study site.

HIV testing of infants born to HIV-infected mothers is undertaken through the established PMTCT program at 6 weeks of age. HIV testing is also undertaken in the majority of children hospitalized to the general wards for severe infections. During 2009, 247 of the estimated 300 HIV-infected infants born in the study area were initiated on triple drug antiretroviral treatment.

Free formula-milk is available to all infants born to HIV-infected mothers at the study site. Although HIV-infected mothers are provided with an option of either exclusively breast-feeding their children or formula feeding, 95% of HIV-infected mothers choose the latter option. As all households have access to running water in the community, the risk related to contamination of formula feeds is consequently minimized. Additionally, whilst breastfeeding is encouraged in HIV-uninfected women, 70% of HIV-uninfected mothers choose to breast feed with the majority of them including additional supplement feeds within three months of age.

In addition to free access to antiretroviral treatment for all HIV-infected individuals based on national treatment guidelines, all HIV-infected individuals also have access to cotrimoxazole prophylaxis. Criteria for initiating cotrimoxazole prophylaxis in HIV-infected mothers include a CD4+ cell count <200 cells/μl. Among infants born to HIV-infected mothers, the site follows the WHO recommendations of cotrimoxazole prophylaxis. This includes, relevant to this study, that prophylaxis be provided to all HIV-infected children under one year of age, irrespective of CD4+ cell count. HIV-infected infants receive their cotrimoxazole prophylaxis through the established HIV care center and prophylaxis is initiated at 4-6 weeks of age in infants born to HIV-infected mothers. Cotrimoxazole prophylaxis is continued in infants born to HIV-infected mothers until the

HIV status of the child is confirmed as being negative either when undertaken at 6 weeks of age in non-breast fed infants, or alternately three months following cessation of breast feeding in breast-fed infants. Based on the low vertical transmission of HIV in the study population (3%) coupled to the majority of infants born to HIV-infected mothers being formula fed, it is expected that approximately 5% of children born to HIV-infected mothers will require cotrimoxazole prophylaxis.

HIV Research: The largest of the HIV research units at CHBH, and one of the largest in South Africa, is the Perinatal HIV Research Unit (PHRU). Dr Violari, a deputy-director in PHRU and collaborator on this study is responsible for overall management of HIV PMTCT in Soweto and at CHBH. A Community Advisory Board (CAB) enables the interactions between the HIV researchers and the community on issues regarding ongoing prevention research, and adult and pediatric therapeutic research. The CAB deals with project specific issues, like assessment of research protocols and informed consent documents, and broader issues surrounding HIV prevention and therapeutic research and assist in community education drives. The RMPRU collaborates extensively with the PHRU in relation to prevention of opportunistic infections, especially in children, including evaluation of different vaccines.

Selection and enrollment of participants

HIV-infected pregnant women will be identified through the PMTCT program and enrolled at CHBH, where annually approximately 18 000 of the 28 500 pregnant women of Soweto will undergo at least one antenatal visit. The overall antenatal attendance rate for at least one visit, including that at primary health care clinics in the area is 99%. Enrolment may also be expanded to other primary health care clinics, including the adjacent Lillian Ngoyi clinic, Mofolo, Diepkloof, Michael Maponya and Chiawelo clinics, at which antenatal services are also offered. Enrolment into the study will occur following screening of mothers for HIV, which is undertaken as part of a routine, well-functioning PMTCT program described above. Specific enrolment inclusion and exclusion criteria are as follows:

Inclusion Criteria

(i) Pregnant women age ≥18 years to <39 years.

(ii) Gestational age ≥12 weeks to <36 weeks documented by the approximate date of the last menstrual period and corroborated by physical/ sonargraphic exam.

(iii) Documented to be HIV-infected on two assays prior to study-enrolment.

(iv) Able to understand and comply with planned study procedures.

(v) Provides written informed consent prior to initiation of study.

Exclusion Criteria

(i) Features of WHO clinical category 3 or 4 of AIDS at the time of enrolment.

(ii) Receipt of TIV, other than through the study, during the current influenza season documented by medical history or record.

(iii) Receipt of any live licensed vaccine ≤28 days or inactivated licensed vaccine (EXCEPT tetanus toxoid) ≤14 days prior to study-vaccine.

(iv) Receipt of a non-licensed agent (vaccine, drug, biologic, device, blood product, or medication) ≤28 days prior to vaccination in this study, or expects to receive another non-licensed agent before delivery unless study approval is obtained.

(v) Any significant (in the opinion of the site investigator) acute illness and/or oral temperature greater than or equal to 38°C ≤24 hours prior to study entry.

(vi) Use of anti-cancer systemic chemotherapy or radiation therapy ≤48 weeks of study enrollment, or has immunosuppression as a result of an underlying illness or treatment.

(vii) Long term use of glucocorticoids, including oral or parenteral prednisone ≥20 mg/day or equivalent for more than 2 consecutive weeks (or 2 weeks total) ≤12 weeks of study entry, or high-dose inhaled steroids (>800 mcg/day of beclomethasone dipropionate or equivalent) ≤12 weeks before study entry (nasal and topical steroids are allowed).

(viii) Receipt of corticosteroids for preterm labor ≤14 days before study entry.

(ix) Receipt of immunoglobulin or other blood products (with exception of Rho D immune globulin) ≤12 weeks prior to enrollment in this study or is scheduled to receive immunoglobulin or other blood products (with the exception of Rho D immune globulin) during pregnancy or for the first 24 weeks after delivery.

(x) Receipt of IL2, IFN, GMCSF or other immune mediators ≤12 weeks before enrollment.

(xi) Uncontrolled major psychiatric disorder.

(xii) History of a severe adverse reaction to previous TIV.

(xiii) Any condition that would, in the opinion of the site investigator, place the subject at an unacceptable risk of injury or render the subject unable to meet the requirements of the protocol.

(xiv) Pregnancy complications (in the current pregnancy) such as pre-term labor, hypertension (BP >140/90 in the presence of proteinuria or BP >150/100, with or without proteinuria or currently on antihypertensive medication) and pre-eclampsia.

Concomitant Medication Guidelines

Administration of any medication, therapies and vaccines will be documented in the study case report forms (CRFs). Concomitant medications will include all medications and vaccines taken during the following periods: 12 weeks prior to enrollment through end of study or early termination, whichever occurs first.

Receipt of any vaccines besides the study product will be collected throughout the study from enrollment to the off study visit. The administration of inactivated licensed vaccines should be at least 14 days prior to the administration of the study vaccine OR delayed until 14 days after the study vaccine has been administered. The administration of live vaccines is contra-indicated during pregnancy. After delivery live vaccines should not be administered ≤28 days of study vaccine administration.

Disallowed/Precautionary Medications

The following medications should be avoided, if possible, and alternative treatments sought. Medications that might interfere with the evaluation of the investigational product should not be used unless absolutely necessary. Medications in this category include, but are not limited to:

Glucocorticoids, i.e. oral, parenteral and high-dose inhaled steroids,

Immunosuppressive or cytotoxic drugs,

IVIG or other IgG preparations other than Rhogam,

Prophylactic use of acetaminophen,

The use of these medications will be recorded throughout the study from enrolment to the off study visit.

Study description and assumptions

Blood samples will be undertaken as shown in the table of procedures.

Humoral immunity will be measured by hemagglutinin antibody inhibition (HAI) assay. In healthy individuals, HAI titers ≥1:10 indicate presence of influenza-specific antibodies and titers ≥1:40 indicate protection against infection and disease. A recent study in HIV-infected children immunized with live, attenuated influenza vaccine (LAIV), reported high HAI titers prior to vaccination were associated with protection against shedding of the vaccine virus [66]. In this study we will use the following definitions to assess the humoral immune response to TIV: HAI titers <1:10 = sero-negative; HAI titers ≥1:10 = sero-positive; HAI titers ≥1:40 = protected against influenza; response to TIV = sero-response, HAI titers ≥1:40 AND ≥4-fold increase in HAI titers from baseline to one month after completion of the dosing schedule.

A pilot study of TIV immunogenicity conducted in 14 HIV-infected and 14 HIV-uninfected pregnant women at the University of Colorado, Denver, USA showed that even before vaccination 75% of both HIV-infected and HIV-uninfected pregnant women had HAI titers ≥1:40 across all vaccine strains and that the median HAI titer was similar in both groups (Fig 1). Administration of TIV generated a significant increase in HAI titers at 6 weeks after vaccination both in HIV-infected and HIV-uninfected women (P=0.02 for both groups, Wilcoxon signed rank test). However HAI titers after vaccination were significantly higher in HIV-uninfected than in HIV-infected women (P=0.007).

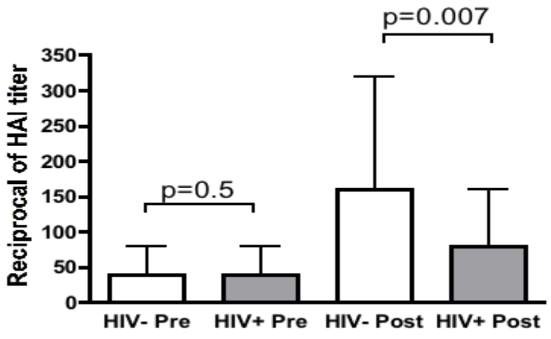

Figure 1. HAI responses to TIV in HIV-infected and HIV-uninfected pregnant women. Data were derived from 28 women immunized with TIV during pregnancy. Sera were collected before and 6 weeks after immunization and antibodies were measured for the 3 serotypes contained in the vaccine. Both HIV+ and HIV- women had significant increases in HAI titers at 6 weeks post- immunization (p=0.02 for both groups), however, HIV- women had more robust antibody responses.

Since TIV is consistently administered to HIV-infected individuals in the USA and somewhat consistently to pregnant women, we assume that some of the baseline HAI titers reflect prior immunization due to HIV or previous pregnancy, in addition to exposure to the wild type virus. In South Africa, TIV is infrequently used and, therefore, we anticipate that baseline HAI titers in South African HIV-infected and HIV-uninfected pregnant women will be lower than those observed in pregnant women in Colorado. In addition, all HIV-infected pregnant women received HAART during pregnancy in the USA, but not yet in South Africa. Since HAART is associated with improved immunogenicity of vaccines, we anticipate that the difference in HAI responses to TIV between South African HIV-infected and HIV-uninfected pregnant women will also be significant and perhaps higher than the difference in responses observed in Denver. We will also assess the effect of TIV administration during pregnancy on transplacental influenza-specific antibody transfer to the fetus. HAI titers, including determining the proportion with titers ≥1:40, will be measured in infants within one week of birth and at 8, 16 and 24 weeks of age.

Cell-mediated immunity (CMI) is an important component of the immune response to influenza. Forrest et al. used ELISPOT to assess CMI responses to influenza vaccines [67]. They found that post-vaccination results ≥100 spot forming centers (SFC)/10^6 peripheral blood mononuclear cells (PBMC) correlated with protection against wild-type infection in children immunized for the first time with LAIV. Less is known about influenza-protective CMI responses after TIV. In a study of healthy adults in Denver, it was found that TIV boosted ELISPOT responses as well as LAIV (Weinberg A: Manuscript in preparation). However, in HIV-infected children neither TIV or LAIV boosted CMI and, furthermore, TIV administration was associated with a decrease of ELISPOT values [68]. Pregnancy has a tolerogenic effect on CMI and, therefore, we anticipate low baseline influenza-specific CMI followed by an increase after vaccine administration. The CMI evaluations in this study will provide novel information on influenza-specific CMI in pregnant women and newborn HIV-exposed infants.

In addition, we will compare the CD4+ cell counts before and after immunization. Blood for CD4+ cell count and HIV viral load will be collected at baseline and within a week of delivery of infant. This is to address concerns raised by the data showing increases in plasma HIV RNA in HIV-infected subjects after immunization and by the STEP study,

which showed a heightened susceptibility of HIV-infection in individuals who received an investigational HIV-vaccine [69]. It is conceivable that the activation induced by TIV is transient and that, in fact, at the end of the influenza season (24 weeks after immunization), the immune system of placebo recipients who were less protected against wild type influenza compared with the vaccine recipients, may have the highest levels of T-cell activation.

Participant enrolment

A prospective study involving 819 (263 in each arm plus 30 in pilot phase in 2012) HIV-infected pregnant women, including follow-up of their infants until 24 weeks of chronological age, will be randomized to receive either

1. Single dose (15µgHA/strain) TIV (SD-TIV),
2. Double dose (30µgHA/strain) TIV on same day (DD-TIV)
3. Two single (15µgHA/strain) doses of TIV 28-35 days apart (2SD-TIV)

Enrolment of HIV-infected women will be targeted at women between 12 to 36 weeks of gestational age. Gestational age at enrollment will be calculated based upon the last known date of menses, clinical evaluation and/ or sonar if available.

This study does not include a placebo arm, although, to maintain blinding, participants will receive at least one dose of a placebo vaccine. All participants will receive two vaccines at visit one, and one vaccine at visit 2 as follows:

1. Single dose (15µgHA/strain) TIV (SD-TIV): Visit 1: TIV and placebo; visit 2: placebo
2. Double dose (30µgHA/strain) TIV on same day (DD-TIV): Visit 1: TWO doses of TIV; visit 2: placebo
3. Two single (15µgHA/strain) doses of TIV 28-35 days apart (2SD-TIV): Visit 1: TIV and placebo; visit 2: TIV

Single dose administration of TIV is recommended in pregnant women, and although it is not yet standard of care, uptake of TIV immunization, particularly in pregnant women has increased in South Africa, especially in the wake of the 2009 pandemic H1N1 outbreak and maternal morbidity and mortality associated with the outbreak. This study will

include the recommended single-dose TIV as a comparator to DD-TIV and 2SD-TIV schedules.

To offset the risk of developing severe influenza illness in study participants, the study will provide oseltamivir to all study participants known to be hospitalized for respiratory illness which is confirmed to be caused by influenza virus. Recommendations on the use of oseltamivir in pregnant women and young infants were recently published by the CDC and the study will use the same recommended doses [70]. It is anticipated that not more than 5% of all study participants will require treatment with oseltamivir.

Sample Size Considerations

The data from a parallel, albeit separate, study on the immunogenicity of TIV in a cohort of HIV-uninfected women and kinetics of transplacental antibody transfer to their infants will be used as a comparator for the immunogenicity and safety of single-dose TIV in HIV-infected women-infant dyads.

The sample size of HIV-infected women to be included in this safety and immunogenicity analysis has been powered at 80% and $\alpha<0.05$ to detect at least a 30% difference in the proportion of HIV-infected mothers who respond to TIV vaccination in arms DD-TIV and 2SD-TIV arms compared to SD-TIV.. An anticipated 44% of women in SD-TIV will have a vaccine response (HAI titers ≥1:40 AND ≥4-fold increase in HAI titers) to the least immunogenic strain in the TIV formulation. In addition, the kinetics of HA antibody will be measured in infants of HIV-infected women within 7 days of birth and at 8, 16 and 24 weeks post-partum.

Sample size calculation (1:1:1 ratio) based on 80% power and alpha less than 0.05.

**Estimated % improvement in sero-conversion**	**Assumed sero-conversion rate**					
	**30%**	**35%**	**36%**	**44%**	**45%**	**50%**
**20%**	1096	865	826	581	557	449
**25%**	715	562	537	376	360	288
**30%**	505	397	**378**	**263**	252	201
**35%**	377	296	**282**	195	186	149
**40%**	294	229	219	151	144	113
**45%**	235	184	175	120	114	90
**50%**	194	151	143	97	92	69

(sample size calculations include an attrition of 10%)

Computer-generated randomization lists, in blocks of 30: 10in each arm (SD-TIV, DD-TIV and 2SD-TIV), will be generated with assignment of a 4-digit study number being done in sequence of enrolment. Block size of 30 was selected for practical and feasible

reasons. Blocks will be allocated consecutively to recruitment site when vaccine/ placebo are requested. Once a block of 30 numbers has been allocated to a recruitment site, the envelopes for allocated block with randomisation forms will be sent to recruitment site by pharmacist with the first set of vaccines prepared for the block. The randomisation forms, in envelopes with printed study number, will be pre-printed with the 4-digit study number and an alphabetical and colour code for vaccine/ placebo. Assignment to an arm of the study will be according to a random computer generated assignment by the study pharmacist. Study medication will be prepared by the pharmacist in syringes filled with either saline or with influenza vaccine as per randomization. Syringes for TIV and placebo administration will be identical to maintain double blinding.

Once an eligible participant has signed informed consent forms, the study doctor/ nurse at recruitment site will select the next randomization envelope (consecutive order), and select a pair of syringes with alphabetical code which corresponds to the pre-printed code on the randomization form. Vaccine/ placebo will be administered by a study-doctor or study-nurse in the deltoid muscle of each arm. Syringes containing TIV and placebo will look identical, however, for visit 1 vaccination; pharmacist will ensure that the syringes are labeled 'dominant' and 'non-dominant'. At visit 1, all participants will receive a dose of TIV in their non-dominant arm. Participants randomized to single dose and 2SD-TIV groups will receive placebo in dominant arm, and participants in double-dose arm will receive a dose of TIV in dominant arm.

At visit 2, all participants will receive an injection in their non-dominant arm: SD-TIV and DD-TIV participants will receive placebo, and 2SD-TIV participants will receive TIV.

Investigational product

In September 2012 WHO recommended a vaccine formulation containing the following influenza strains:

- An A/California/7/2009 (H1N1)pdm-like virus

- An A/Victoria/361/2011 (H3N2)-like virus

- An B/Wisconsin/1/2010-like virus. (Yamagata lineage).

TIV formulation: 0.5ml dose includes 15µg haemagglutinin (HA) of each of the 3 strains.

Preparation of Blinded Study Products

TIV

Using aseptic technique, 0.5 mL of TIV will be injected from a pre-filled syringe into a sterile 2ml syringe. A sterile needle for administration of TIV will be secured to the syringe. The study product will be labeled with one of five alphabetical letters assigned to TIV.

PLACEBO

Using aseptic technique, 0.5 mL of 0.9% NaCl solution will be drawn into a single-dose 2ml size syringe. The placebo will be labeled with one of the alphabetical letters assigned to placebo.

The study statistician, pharmacist and unblinded dispensing doctor will be the only members of investigative team who will be able to match alphabetical codes to TIV or placebo.

Product Supply, Distribution and Pharmacy

Study Product Acquisition and Distribution

TIV and 0.9% NaCl will be procured directly by RMPRU through the study grant from the local distributor of the vaccine. Purchase of TIV will be managed by the site pharmacist/dispenser or study coordinator. The study site pharmacists/dispenser will maintain complete record of all study products procured and dispensed. Sites will receive instructions regarding the final disposition of any remaining study products.

Subject follow-up**:**

The study will undertake active surveillance as detailed. The criteria for investigating for influenza illness will be as follows:

Screening for possible influenza illness and other serious adverse events will include weekly home visits or telephonic contact of study participants from the time of enrolment of the mother. Home surveillance visits and or telephonic interviews will be undertaken by trained field workers, assisted by trained nurses, using structured questionnaires to interview for symptoms. The RMPRU has had previous experience in undertaking weekly home-visits as part of screening for illnesses during previous vaccine-interventional trials.

In addition, screening of hospital admission books to identify participants who have been admitted to hospital with a respiratory or other illness will be undertaken. Participants with influenza-like illness will have respiratory samples collected. Participants attending study center for unsolicited illness visits will be investigated for Influenza. In addition, participants will be sent weekly short message service (SMS) text as reminders of the symptoms of influenza illness with a request to attend the clinic if the mother or child fulfills pre-specified criteria suggestive of influenza illness.

Mothers will also be trained how to measure the child's axillary temperature and their oral temperature using temperature probes to be supplied by the study whenever suspecting fever.

Sample collection for influenza virus testing will involve nasopharyngeal aspirates (NPA) from infants and nasopharyngeal (NP) flocked swabs and oropharyngeal (OP) swabs from the mothers. The site has established standard operating procedures for these methods and has undertaken NPA in previous studies on respiratory viruses in children. Although the yield between oropharyngeal swabs and NPA is similar for influenza virus in children, NPA has the advantage of obtaining samples which can be explored for other respiratory viruses as well.

Investigating for influenza virus infection

The proposal plans on having a low threshold for investigating for influenza illness, as the signs and symptoms may be very non-specific, especially in very young infants. Additionally, the study will collect information of any symptom and signs that may be suggestive of influenza illness, to allow for analysis of influenza virus associated with a specific group of symptoms and signs. The final case definition of ILI and grading of influenza severity for infants and mothers will be done, a priori, during the development of the analysis plan. This will allow for consultation with other investigators from other countries where similar studies may be undertaken during the course of this study.

Criteria for investigating for influenza illness in infants

(i) Any documented fever (≥37.8°C on axillary measurements for <7 days duration) irrespective of presence of respiratory symptoms and signs;

(ii) Fever (documented as ≥37.8°C and/ or mother's perception that infant feverish/ hot) plus at least one sign/ symptom of acute respiratory infection (see point iii below) WITHIN THE PAST 72 HOURS;

(iii) At least TWO signs/ symptoms of ARTI WITHIN THE PAST 72 HOURS

- Tachypnoea (RR≥60 breaths /min in infant 0-2 months of age; RR≥50 br/min in infant 2-12 months of age)
- Difficulty breathing (reported by mother: noisy/ interrupted/ irregular/ or fast)
- Coughing
- Wheezing
- Runny or congested nose
- Cyanosis/ O_2 saturation < 90% (if available)
- Chest wall indrawing
- Grunting on expiration
- Pus draining from ear

(iv) Hospitalization for any severe illness or any respiratory illness (of presumed infectious origin) with symptom onset of <7 days duration (including children fulfilling WHO criteria of severe pneumonia/ very severe disease);

(v) History of apnea within past 72 hours in the infant;

(vi) Any unsolicited illness visit to the clinic with respiratory symptoms of <7 days duration.

Note: Detailed information of signs and symptoms will be collected on all children in whom samples are taken for influenza testing.

<u>*Criteria for investigating for influenza in mothers*</u>

(i) Any hospitalization for an acute respiratory illness of <7 days duration;

(ii) Presence of fever (≥38 °C on oral measurements) or chills/ rigors or feeling feverish in past for <7 days duration OR;

(iii) AND ONE of following for <7 days duration:

a. cough or sore throat or pharyngitis,

b. muscle aches or joint aches or headaches,

e. chest pain while breathing or feeling short of breath or had difficulty breathing.

(iv) AND the absence of other diagnosis causing these symptoms.

(v) All participants attending clinic for unsolicited respiratory illness visits with at least one respiratory symptom/ fever documented in previous 7 days, irrespective of whether or not they fulfill ILI definition.

(vi) Alternatively, in the absence of fever and chills/rigors, the presence of symptoms from at least 2 of the remaining three groups of symptoms, in the absence of another diagnosis will require testing for influenza virus.

Samples for influenza virus testing should ideally have been obtained within 96 hours of symptom onset and no later than 7 days after initial onset of symptoms.

Influenza virus identification and molecular characterization

Viral particles will be detected in the NP/OP swabs and/or the NPA by a qualitative two step real time reverse transcriptase-polymerase chain reaction (rRTPCR) assay. Total nucleic acids will be extracted from 200µl of sample with a Biomerieux Nuclesin nucleic acid extraction robot (Biomerieux). Nucleic acids will be eluted in the standard elution buffer and stored at -70°C. Complementary DNA (cDNA) will be synthesized by using MultiScribe reverse transcriptase (RT) and random hexamers (both from Applied Biosystems). Reactions will be performed with 5µl of sample in a final volume of 20µL according to the manufacturers' recommendations. After incubation for 10 minutes at 25°C, RT is carried out for 30 minutes at 48°C, followed by RT inactivation for 5 minutes

at 95°C. cDNA will be stored at -20°C. Primers and probe sets which target either the matrix gene or the hemagglutinin gene designed for the universal detection of type A and B influenza viruses, respectively, will be used in the PCR assays. Each sample will also be tested for a human positive control gene (Glyceraldehyde-3-phosphate dehydrogenase) to account for possible extraction and assay set up errors. All influenza A viruses will be further subtyped as either H1 or H3 using primers and probe sets that target the hemagglutinin genes of each virus. Testing for influenza virus will be undertaken at the RMPRU with quality assurance done at NICD. The choice of rRTPCR instead of viral culture for influenza virus is based on its increased sensitivity. rPCR typically detects an excess of 20% positive influenza specimens compared to viral culture. Both methods have equal specificity. The rPTPCR is performed under rigorous good laboratory practice conditions and the risk of contamination is minimal. The PCR targets are conserved areas of influenza A and influenza B genes and, therefore, we anticipate that antigenic drifts and shifts will not affect its diagnostic accuracy. Evaluation for genetic drift of influenza virus will be undertaken at NICD by sequencing of the HA1 subunit of the hemagglutinin and neuraminidase genes.

Samples obtained from infants and mothers will be archived at -70°C to examine for other respiratory viruses which may also contribute to respiratory illness. Funding for this aspect of the study will be sourced through alternate avenues. The specific methods used for influenza and other respiratory virus testing are included in a specific laboratory Standard Operating Procedure manual.

Breast-feeding practices

Breast-milk will be collected from breast-feeding mothers to assess the level of HAI IgA titers. Information on feeding practices will be collected at scheduled and weekly visits.

Immunogenicity studies

Humoral and cell-mediated immune (CMI) responses to influenza strains in the vaccine will be measured to assess the immunogenicity of TIV in pregnant women and their infants.

Hemagglutination inhibition assays will be performed on serum or plasma as described previously [71]. Briefly, specimens are treated with receptor-destroying enzyme (RDE) by diluting one part sample with three parts enzyme and incubating overnight in a 37°C water bath. The enzyme is inactivated by 30-min incubation at 56°C followed by addition of six parts 0.85% physiological saline for a final dilution of 1/10. HAI assays are performed in either V-bottom 96-well microtiter plates with 0.5% turkey erythrocytes, or in U-bottom 96-well microtiter plates with 0.5% guinea pig erythrocytes. Sera will be titrated against antigens from the influenza vaccine strains A H1N1 California, A H3N2 Perth and B Brisbane.

The importance of cytotoxic T lymphocytes (CTL) as a major contributor to defense mechanisms against influenza in humans has long been recognized [78]. In peripheral blood mononuclear cells (PBMC) from adults, influenza-specific memory CTL can be stimulated to rapidly develop effector function [79]. Granzyme B (GrzB) is a proapoptotic serine protease released from CTLs into influenza-infected target cells through a process facilitated by perforin, GrzB are transported across the cell membrane into the cytoplasm of the infected host cell. In animal models GrzB activity was reported to be the earliest and main contributor to CTL-mediated killing of influenza-infected cells [80]. GrzB is a key element in the enzymatic cascade that leads to apoptotic cell death and clearance of influenza virus from the lungs [80]. It has been shown that in older adults, GrzB activity in influenza-challenged PBMC correlates with protection against influenza and illness severity and that prior to influenza illness GrzB levels are lower in the influenza-stimulated PBMC from adults who subsequently develop influenza illness compared to those who do not [81,82]. Correlates of protection based on the cell-mediated immune response may complement antibody responses in screening for the best vaccine candidates. GrzB activity will be measured in lysates of influenza-stimulated PBMC.

Interferon (IFN)-□ ELISPOT responses will be assessed on frozen PBMCs as previously described [51]. Briefly, PBMCs will be separated with Ficoll-Hypaque gradients and stimulated for 16–20 h *in vitro* with 10 TCID50/cell of attenuated monovalent influenza virus corresponding to the vaccine strains (A H1N1 California, A H3N2 Perth and Brisbane); and with medium and phytohemagglutinin (PHA) (5 mg/ml) as controls. Spots will be visualized with a CTL ELISPOT plate reader. Background (non-specific) spots detected in the medium-containing wells will be subtracted from the wells stimulated with influenza antigens. Results will be reported as SFC/10^6 PBMCs.

The study will assess the effect of TIV administration during pregnancy on transplacental influenza-specific antibody transfer to the fetus. HAI titers will be measured in infants within one week of birth and at 8, 16 and 24 weeks of age. Using the 1:40 HAI titer as a defining threshold of protection against wild type influenza, we will determine the proportion of infants protected against influenza at birth and the duration of the protection conferred by transfer of maternal antibodies. We anticipate a higher proportion of protected infants among those born to vaccinated mothers compared with placebo recipients.

Processing of MATERNAL and INFANT samples

HAI assays. HAI titers against each strain in the vaccine will be measured on blood samples obtained from women at baseline, at 28-35 days post-first vaccination, 28-35 days post-second vaccine dose, at delivery (±1 week) and 24 weeks post-partum. HAI will be performed as described previously [71]. The antigens used in these assays will match the seasonal influenza vaccine serotypes obtained from the USA CDC. Detail SOPs for the assay are available from Dr. Weinberg's laboratory where assays will be undertaken. The kinetics of HA transferred to infants will be measure by examining HAI titers within the first week of life and subsequently at 8, 16 and 24 weeks of age.

CMI assays will be established in South Africa. ELISPOT to determine CMI responses on vaccinated women from bloods drawn prior to and 4 and 24 weeks post vaccination and in infants from bloods within 7 days of birth, against the strains of influenza contained in the vaccine will be primarily assessed using an established assay at Dr.

Madhi's laboratory where personnel will be trained by Dr. Weinberg as previously done elsewhere [72].

T-lymphocyte activation assays will be performed in Dr. Weinberg's laboratory using state-of-the art polychromatic flow cytometry. The T- and B-cell phenotypes are assessed by flow cytometry using freshly thawed PBMC. Cells are stained using monoclonal antibodies against the following molecules: CD3 (BD Biosciences), CD4 (Beckman Coulter), CD19 (BD Bioscience), IL-10 (eBioscience), TGFβ (Cedarlane), FoxP3 (eBioscience), CD25 (BD Biosciences), CD38 (BD Biosciences) and HLADR (BD Biosciences). Total lymphocytes and subpopulations are counted on Guava easyCyte 8HT (Millipore) and analyzed with FlowJo (Treestar). Subpopulations are expressed as a percentage of the parent CD4+, CD8+ or CD19+ cell population.

HIV viral load, HIV-ELISA and CD4+ testing will be undertaken by the National Institute of Health accredited Contract Research Laboratory Services in Johannesburg.

Nasopharyngeal Streptococcus pneumoniae colonization studies: NP specimen will be collected from the infants using Dacron swabs that will be placed in skim milk tryptone-glucose-glycerin (STGG) medium. Carriage will be measured by culture as well as by quantitative molecular analysis of pneumococcal density using real time PCR for the lytA gene and molecular analysis of serotypes present.

Nasopharyngeal viral carriage studies: Flocked NP and OP specimens will be collected from all infants at scheduled visits 4, 5 and 6 and placed in viral transport medium (VTM). Detection of different respiratory virus by PCR will be performed in these samples at a later stage.

Interaction between Streptococcus pneumoniae and influenza virus: We will explore colonization by S. pneumoniae at the time of influenza-like illness in mothers and children to explore the idea that there may be a quantitative cutoff of S. pneumoniae colonization in the nasopharynx that is predictive of pneumococcal disease. Additionally, individuals with febrile illness will be investigated for *S. pneumoniae* co-infections by lytA PCR on serum or whole blood [73]. Although most infants in this study will receive PCV at 6 and 14 weeks of age, the overall effect of PCV immunization against pneumococcal

disease in the target population of children under 24 weeks of age, and particularly those less than 16 weeks of age is expected to be modest considering that optimal protection is only likely to be apparent at least two weeks after the second dose of PCV. Additionally, a 9-valent PCV vaccine, as opposed to a 7-valent vaccine currently used was associated with only 35% reduction of all invasive pneumococcal disease in HIV-infected children. Consequently, despite immunization with a 7-valent PCV, there is still likely to be a high residual burden of pneumococcal disease in the study population.

Evaluation of the role of S. pneumoniae co-infection in children and mothers with ILI and confirmed influenza illness using standard culture methods of blood and sputum (mothers only), lytA PCR and nasopharyngeal (or oropharyngeal in mothers) swabs analyzed by routine culture and quantitative lytA PCR [74].

Participant Management

Toxicity Management

It is anticipated that vaccine-associated adverse events (AEs) will occur frequently, but that these will be minor local reactions and side effects. All AEs will be managed appropriately according to the situation.

Toxicities will be classified by the Division of AIDS Table for Grading the Severity of Adult and Pediatric Adverse Events, Version 1.0, dated December 2004, (Clarification dated August 2009), which is available on the web (http://rsc.tech-res.com/safetyandpharmacovigilance).

A clinic visit will be required within 72 hours for maternal vaccine-related (in the opinion of the investigator) adverse reactions Grade ≥3. Other Grade <3 reactions will be managed as per standard of care.

Examples of Adverse Events of Grade ≥ 3 that may be related to the vaccines in the absence of an alternative explanation:

i. Abnormal laboratory values, signs and symptoms or diagnoses.

ii. Local AEs, including pain, tenderness, redness, and swelling post vaccination.

iii. Systemic AEs, including feverishness, malaise, body aches (exclusive of the injection site), nausea, and headache post vaccination.

Iv. Adverse pregnancy outcomes, including maternal, fetal and infant complications.

ALL Grade ≥2 maternal adverse events regardless of association to vaccine will be recorded on the appropriate CRF.

Infant Grade ≥3 adverse events will be reported on the appropriate CRF. Management of the infant will be as per local standard of care.

Participant Management

Screening and study entry may occur on the same day; however, entry may be delayed up to 28 days after screening, if required.

Maternal history at screening/entry should include all history of cancer, history of all immunizations received within the past 12 weeks, status of current pregnancy and history of previous pregnancy complications and all medications taken within the past 28 days. After entry, cancer, allergies, all pregnancy related diagnoses, current medications, as well as any incidence of possible influenza illness (pneumonia, upper respiratory tract infection or other lower respiratory infection) should be reported. All diagnoses of congenital anomalies will be reported for the infant.

Physical exam at screening/entry should include vital signs (temperature, blood pressure, heart rate, and respiratory rate), weight and complete physical exam as per local standards. After entry, physical exam should include vital signs, weight, and targeted exam based on current signs and symptoms.

Mothers will remain in the clinic for at least 30 minutes after vaccination so that clinic personnel can observe for any potential adverse reactions to the vaccine. Equipment, supplies, and properly skilled medical personnel must be immediately available for emergency use in the event of an unexpected adverse reaction.

Report of vaccine-related local (redness, swelling, tenderness, itching) and systemic (fever, malaise, myalgia, nausea, headache, rash) adverse events will be solicited at day 7 and day 28. Serious Adverse Events (SAEs) will be reported as described below.

Antipyretics should not be routinely given in anticipation of adverse events after vaccination, but should not be withheld if symptoms occur. Antipyretics should be recorded on the memory aid and reported to the site staff. Breastfeeding is permitted in this study and the information recorded.

Infants will receive all the vaccine included the South African Expanded Program for Immunization as per standard of care. Infant AEs will be managed as per local standard of care. Infant Grade ≥3 AEs will be recorded at each study visit for the period of time elapsed since the previous visit.

Criteria for Study Discontinuation

The subject or legal guardian refuses further treatment and/or follow-up evaluations.

The investigator determines that further participation would be detrimental to the subject's health or well-being.

The subject fails to comply with the study requirements so as to cause harm to him/herself or seriously interfere with the validity of the study results.

SAE Reporting Requirements for this Study

The SAE for which expedited reporting (within 24 hours of site awareness thereof) are required for this study will include: maternal death during pregnancy and after delivery. During pregnancy, mothers will receive TIV or placebo in a blinded fashion. SAE will be reported without knowledge of treatment arm. At the end of the study, when the treatment groups will be known to the study team, SAE will be attributed to one of the active products or to placebo.

In addition to the SAE Reporting Category identified above, other AEs that must be reported in an expedited manner are: maternal hospitalizations other than for delivery, fetal loss, miscarriages, congenital anomalies, infant death through 24 or 28 weeks of life, whichever coincides with the end of the study follow up.

Grading Severity of Events

The Division of AIDS Table for Grading the Severity of Adult and Pediatric Adverse Events, Version 1.0, dated December 2004, (Clarification dated August 2009), is used and is available on the RSC web site (http://rsc.teches.com/safetyandpharmacovigilance).

Expedited AE Reporting Period

The expedited AE reporting period for this study is the entire study duration for an individual subject (from study enrollment until study completion or discontinuation of the subject from study participation for any reason).

Monitoring

Safety and tolerability of the study vaccine will be monitored by means of AEs and toxicity reports presenting laboratory and clinical data. All participants will complete a

dairy card for the week following each vaccination visit. A pilot study including 30 women randomized 1:1:1 to single, double and two-dose arms has been completed. Due to the potentially more severe reactions in the double-dose arm, the Data and Safety Monitoring Board (DSMB) reviewed solicited local and general reactions data stratified for treatment arm after the first 30 participants (10 in each group) had been enrolled. Enrollment was halted while the DSMB reviewed and approved continuation of single, double and two-dose treatment arms. At the DSMB and investigators meeting in November 2012, the DSMB expressed no safety concerns, and approved trial continuation with single, double and two-dose arms.

The data to be reviewed by the protocol team will be pooled across treatment arms. It is required that these data be entered into the database within 48 hours of the time at which the results of the laboratory tests or clinical examinations become available. These reports will be discussed by the Core Team (comprised of the Study Chair and other co-investigators as well as the data manager and study-statistician) on monthly conference calls. Interpretation of vaccine-relationship to AEs will be based on the type of event, the relationship of the event to the time of vaccine administration, the known biology of the vaccine and the investigators' medical judgment. A vaccine-related AE refers to an AE for which there is a possibly, probably or definite relationship to the administration of the vaccine. The investigators determine the relationship to the vaccine using the following definitions: a) Unable to judge; b) Not related; c) Probably not related; d) Possibly related; e) Probably related; f) Definitely related.

In addition to monthly toxicity reviews by the Core Team, the study will be monitored by the DSMB. DSMB members will be independent of the study (except for the statistician) and have no perceived conflict of interest. The committee will meet annually either via conference call or face-to-face to review relevant data. The Chair of the DSMB will be mandated to report the DSMB's comments to the Gates foundation and Protocol Team.

Early stopping rules for safety

The following stopping rules will be applied in order to protect the study subjects from unnecessary exposure to the vaccine, should the safety profile prove unacceptable in this population.

If at any time a subject experiences a life-threatening grade 4 toxicity, judged to be at least possibly related to the vaccine, the Core Team will request an independent review of the data by the DSMB.

If 20 or more subjects have experienced non-life-threatening grade 3 or 4 toxicities, judged to be at least possibly related to study treatment, then the study statistician will unblind the treatment assignments and summarize toxicities occurring in the subjects receiving placebo and those receiving TIV. In collaboration with an independent safety monitor who is a member of or appointed by the DSMB, the statistician will inform the study-team whether ≥30% of the subjects receiving either of the vaccine products have experienced non-life-threatening grade 3 or 4 toxicities which are at least possibly vaccine-related. If this has occurred, the study-team will request an independent review of the data by the DSMB. Otherwise, enrollment will continue.

Since unanticipated serious adverse reactions are not expected, enrollment will continue while the early safety review is taking place.

If the DSMB decides to halt the study for safety reasons and notifies the Study Team, the Study Team will notify the Gates Foundation, regulatory authorities and relevant stake holders immediately.

Human subjects protection: Institutional Review Board and Informed Consent

This protocol, the informed consent document, and any subsequent modifications must be reviewed and approved by the Human Research Ethics Committee (HREC) of the University of the Witwatersrand that is responsible for oversight of the study. Written informed consent must be obtained from the subject (mother). The informed consent will describe the purpose of the study, the procedures to be followed, and the risks and benefits of participation. A copy of the consent form will be given to the participant. Should the consenting parent demise during the course of follow-up of the infant, the other parent of the child if available will be requested to consent for the continued participation of the child in the study. Should the mother have been the consenting parent and the father of the child be unavailable for consenting and not involved in the care of the child, the person taking on the role of primary care-giver to the child will be informed of the study. The continued participation of the infant will depend upon agreement thereof by the new primary care-giver, as indicated by signed consent, and in conjunction with notification thereof on a case-by-case basis to the HREC.

Subject Confidentiality
All laboratory specimens, evaluation forms, reports, and other records will be identified only by a coded number to maintain subject confidentiality. All records will be kept in a secured area. All computer entry and networking programs will be done with coded numbers only. Clinical information will not be released without written permission of the subject, except as necessary for monitoring by regulatory authorities or their designates.

Publication of Research Findings

Any presentation, abstract, or manuscript will be made available for review by the core study-team prior to submission. A detailed publication policy will be developed during the course of the study.

Biohazard Containment

As the transmission of HIV and other blood borne pathogens can occur through contact with contaminated needles, blood, and blood products, appropriate blood and secretion

precautions will be employed by all personnel in the drawing of blood and shipping and handling of all specimens for this study, as currently recommended by the Centers for Disease Control and Prevention.

All infectious specimens will be sent using the ISS-1 SAF-T-PAK mandated by the International Air Transport Association Dangerous Goods Regulations-Packing Instruction 602. Refer to individual carrier guidelines (e.g., Federal Express or Airborne) for specific instructions.

Protection of staff: Because of the increased risk of influenza virus exposure by study-staff dealing with ill participants, special efforts will be undertaken to minimize the risk of influenza illness in staff. This will include training of staff in collecting illness-visit samples investigating for influenza, using standard operating procedures including the wearing of disposable gloves and the use of surgical masks. Additionally, all study-staff will be offered TIV vaccination and oseltamivir will be provided to any staff-member developing severe influenza illness during the course of the study. These measures will be further elaborated on in a SOP of managing staff with influenza-like illness.

Analysis

Safety

For each group of mothers, the proportion of subjects who experience grade 3+ adverse events during the study will be presented and bounded by 90% confidence intervals. This will provide 95% confidence that the true population proportion is no higher than the upper limit of the interval. The proportion experiencing grade 3+ adverse events judged to be treatment-related will also be shown. Since relatively few events of this severity are expected these analyses will be descriptive. However, should an unexpected epidemic of respiratory, CNS, febrile or exanthemous illness occurs during the study, there will more adverse events and a more formal analysis will be performed. In this case, the inclusion of the comparator group (single-dose TIV) will allow us to examine whether the symptoms associated with these adverse events are significantly more prevalent among participants receiving double or two-doses of TIV than among single dose TIV recipients and should attributed to the vaccination dosing schedule.

Immunology

In the mother:

(i) Proportion of subjects with HAI titer ≥1:40 for each strain in the vaccine will be compared SD-TIV vs. DD-TIV and SD-TIV vs. 2SD-TIV and before and after vaccination.

(ii) Comparison of the proportion of subjects with ≥4-fold increase in HAI titer after vaccination compared to baseline for each strain in the vaccine for each study group.

(iii) Composite proportion of subjects with HAI titer ≥40 and 4-fold increase in HAI titer after vaccination for each strain in the vaccine per study group.

(iv) Comparison of the geometric mean HAI titers for each strain in the vaccine before and after immunization.

(v) Comparison of ELISPOT-measured SFC for each strain in the vaccine at baseline and after vaccination.

(vi) Comparison of the proportion of responders defined by an ELISPOT result ≥50 SFC/10^6 PBMC and ≥2-fold increase over baseline.

(vii) Comparison of the percentages of circulating regulatory B and T cells characterized by production of IL-10 or TGFβ, or by expression of FoxP3.

In the infant:

(i) Proportion of infants with cord blood (1st week of life) and at 8, 16 and 24 post-partum with HAI titer ≥1:40 for each strain in the vaccine.

(ii) Comparison of the geometric mean HAI titers for each strain in the vaccine.

(iii) Comparison of the maternal/fetal HAI titer ratio for each strain in the vaccine.

(iv) Proportion of infants with an ELISPOT result ≥50 SFC/10^6 PBMC in first week of life.

Primary immunogenicity analysis

Maternal:

To compare the maternal TIV response rates at 28 days after each immunization to each of the three influenza vaccine strains (H3N2, H1N1, influenza B), between the study

groups, the Chi-square test will be used. Logistic regression analysis may also be performed, should the need arise to control for certain covariates.

Infant:

Chi-squared tests will be used to compare the proportion of infants meeting the primary criterion for seroprotective levels of antibody to each of the three vaccine strains within 7 days of birth. Logistic regression analysis may also be performed, should the need arise to control for certain covariates.

Secondary immunogenicity analyses

Similar analyses to the primary analysis discussed above, comparing persistence of seroprotective levels of HA antibody to individual vaccine strains between infants of mothers in the three vaccine groups will be undertaken 8, 16 and 24 weeks.

In the mothers also similar analyses to the primary analysis will be done comparing the three TIV groups with respect to immunologic response at other time points will be performed.

Efficacy

The secondary analysis of efficacy of TIV will be analyzed as per intent-to-treat protocol. This will involve all events occurring in mother and/or infant-pairs from the date of receipt of the study vaccine. A “per protocol” analysis on efficacy which will be limited to inclusion of HIV-infected mothers who received the correct randomized study-group within the study-defined window periods, the infant was born at ≥37 weeks of gestational age or birth weight of ≥2 500 g, and birth occurred at least 28 days after the mother had received study vaccine.

Efficacy will be measured using the following formula:

$$VE(\%)=\frac{(\text{incidence rate in the single dose group-incidence rate in the double dose group})}{\text{incidence rate in the single dose group}}\times 100$$

$$VE(\%)=\frac{(\text{incidence rate in the single dose group-incidence rate in the two doses group})}{\text{incidence rate in the the single dose group}}\times 100$$

Only the first of any specified event being analyzed will be included in the analysis for any participant.

Specific TIV efficacy endpoint outcomes will include:

In the infant:

(i) Laboratory-confirmed influenza illness in the infant from birth until 24 weeks chronological age.

(ii) Clinically diagnosed protocol defined influenza-like illness in the infant from birth until 24 weeks chronological age.

In the Mother:

(i) Laboratory-confirmed influenza illness in the mother from birth until 24 weeks post-partum.

(ii) Clinically diagnosed protocol defined influenza-like illness in the mother from birth until 24 weeks post-partum.

(iii) The composite of influenza confirmed and/or clinically diagnosed protocol defined influenza-like illness in the infant from birth until 24 weeks chronological age.

Appendix 1: Schedule of Events

	Visit 1A (enrollment)	Visit 1B	Visit 2	Visit 3	Visit 4	Visit 5	Visit 6	Weekly contact	Illness visit
Gestational age (weeks)/ Age	≥12- <36	1 month post visit 1 (28-35 days)	1 month post visit 2 (28-35 days)	Delivery / (0-7 days)	8 weeks post delivery (56-63 days)	16 weeks post delivery (112-119 days)	24 weeks post delivery (168-175 days)		
ICF signed	X								
Inclusion/ exclusion/ Withdrawal criteria	X	X	X	X	X	X	X		
Medical history (Mom)	X	X	X	X			X		X
Targeted physical exam (Mom)	X	X	X	X			X		X
Maternal blood draw for HAI	X	X	X	X			X		
Maternal blood for CD4+, HIV viral load, CD4+ activation	X			X					
Maternal blood for CMI (purple top)	X	X	X				X		
Breast milk collection in breast feeding mothers				X	X	X	X		
TIV/ placebo administered	X (2-vaccines)	X (1 vaccine)							
Diary card dispensed	X	X							
Diary card collected		X	X						
Local/ systematic reactions	X	X (reviewed)	X (reviewed)						
Obstetric outcomes				X					
Newborn/ infant assessment, physical examination				X	X	X	X		X
Medical history (infant)				X	X	X	X		X
Infant blood for HAI				X	X	X	X		
Infant blood for CMI				X			X		
Infant NP and OP swab for					X	X	X		

viral detection									
Infant NP swab for S. pneumo & S. aureus‡					X	X	X		

	Visit 1A (enrollment)	Visit 1B	Visit 2	Visit 3	Visit 4	Visit 5	Visit 6	Weekly contact	Illness visit
Questionnaire re symptoms								X	
Concomitant medication (Mom only X or Mom & baby XX)	X	X	X	XX	XX	XX	XX		XX
Report/ Assess AE & SAE	X	X	X	XX	XX	XX	XX		XX
Maternal NP and OP swab for S. pneumo colonisation									X
Serum for S. pneumo lytA PCR (Mom)									X
Flocked swab and OP swab on mother for influenza									X
Sputum collection from mother (S. pneumo)*									X
Infant NP swab for S. pneumo colonisation									X
NPA on infants for influenza									X
Serum for S. pneumo lytA PCR (Infant)									X

* To be collected in hospitalized cases of pneumonia / bronchiolitis

Reference List

1. Neuzil KM, Mellen BG, Wright PF, Mitchel EF, Jr., Griffin MR. The effect of influenza on hospitalizations, outpatient visits, and courses of antibiotics in children. N Engl J Med 2000;342:225-31.
2. Walter EB, Englund JA, Blatter M, Nyberg J, Ruben FL, Decker MD. Trivalent inactivated influenza virus vaccine given to two-month-old children: an off-season pilot study. Pediatr Infect Dis J 2009;28:1099-104.
3. Halasa NB, Gerber MA, Chen Q, Wright PF, Edwards KM. Safety and immunogenicity of trivalent inactivated influenza vaccine in infants. J Infect Dis 2008;197:1448-54.
4. Munoz FM, Englund JA. A step ahead. Infant protection through maternal immunization. Pediatr Clin North Am 2000;47:449-63.
5. Mak TK, Mangtani P, Leese J, Watson JM, Pfeifer D. Influenza vaccination in pregnancy: current evidence and selected national policies. Lancet Infect Dis 2008;8:44-52.
6. Halling SF, Soderberg MP, Berg UB. Henoch Schonlein nephritis: clinical findings related to renal function and morphology. Pediatr Nephrol 2005;20:46-51.
7. Naleway AL, Smith WJ, Mullooly JP. Delivering influenza vaccine to pregnant women. Epidemiol Rev 2006;28:47-53.
8. Tong A, Biringer A, Ofner-Agostini M, Upshur R, McGeer A. A cross-sectional study of maternity care providers' and women's knowledge, attitudes, and behaviours towards influenza vaccination during pregnancy. J Obstet Gynaecol Can 2008;30:404-10.
9. Zaman K, Roy E, Arifeen SE, et al. Effectiveness of maternal influenza immunization in mothers and infants. N Engl J Med 2008;359:1555-64.
10. 2008 National Antenatal Sentinel HIV & Syphilis Prevalence Survey, South Africa. Department of Health Available at: http://wwwdohgovza/docs/ 2009.
11. Prevention and control of influenza. Recommendations of the Immunization Practices Advisory Committee (ACIP). MMWR Recomm Rep 1992;41:1-17.
12. Neuzil KM, Coffey CS, Mitchel EF, Jr., Griffin MR. Cardiopulmonary hospitalizations during influenza season in adults and adolescents with advanced HIV infection. J Acquir Immune Defic Syndr 2003;34:304-7.
13. Radwan HM, Cheeseman SH, Lai KK, Ellison IR. Influenza in human immunodeficiency virus-infected patients during the 1997-1998 influenza season. Clin Infect Dis 2000;31:604-6.
14. Nabeshima S, Ariyama I, Chong Y, et al. Influenza in three patients with human immunodeficiency virus infection. Intern Med 2000;39:592-7.
15. Fine AD, Bridges CB, De Guzman AM, et al. Influenza A among patients with human immunodeficiency virus: an outbreak of infection at a residential facility in New York City. Clin Infect Dis 2001;32:1784-91.
16. Golden MP, Sajjad Z, Elgart L. Influenza and human immunodeficiency virus infection: absence of HIV progression after acute influenza infection. Clin Infect Dis 2001;32:1366-70.
17. Madhi SA, Schoub B, Simmank K, Blackburn N, Klugman KP. Increased burden of respiratory viral associated severe lower respiratory tract infections in children infected with human immunodeficiency virus type-1. J Pediatr 2000;137:78-84.
18. Madhi SA, Ramasamy N, Bessellar TG, Saloojee H, Klugman KP. Lower respiratory tract infections associated with influenza A and B viruses in an area with a high prevalence of pediatric human immunodeficiency type 1 infection. Pediatr Infect Dis J 2002;21:291-7.
19. Rich KC, Siegel JN, Jennings C, Rydman RJ, Landay AL. Function and phenotype of immature CD4+ lymphocytes in healthy infants and early lymphocyte activation in uninfected infants of human immunodeficiency virus-infected mothers. Clin Diagn Lab Immunol 1997;4:358-61.

20. Clerici M, Saresella M, Colombo F, et al. T-lymphocyte maturation abnormalities in uninfected newborns and children with vertical exposure to HIV. Blood 2000;96:3866-71.
21. Kuhn L, Meddows-Taylor S, Gray G, Tiemessen C. Human immunodeficiency virus (HIV)-specific cellular immune responses in newborns exposed to HIV in utero. Clin Infect Dis 2002;34:267-76.
22. Van Rie A, Madhi SA, Heera JR, et al. Gamma interferon production in response to Mycobacterium bovis BCG and Mycobacterium tuberculosis antigens in infants born to human immunodeficiency virus-infected mothers. Clin Vaccine Immunol 2006;13:246-52.
23. Invasive pneumococcal disease in young children before licensure of 13-valent pneumococcal conjugate vaccine - United States, 2007. MMWR Morb Mortal Wkly Rep 2010;59:253-7.
24. Mansoor N, Abel B, Scriba TJ, et al. Significantly skewed memory CD8+ T cell subsets in HIV-1 infected infants during the first year of life. Clin Immunol 2009;130:280-9.
25. Madhi SA, Adrian P, Cotton MF, et al. Effect of HIV infection status and anti-retroviral treatment on quantitative and qualitative antibody responses to pneumococcal conjugate vaccine in infants. J Infect Dis 2010;202:355-61.
26. Tasker SA, O'Brien WA, Treanor JJ, et al. Effects of influenza vaccination in HIV-infected adults: a double-blind, placebo-controlled trial. Vaccine 1998;16:1039-42.
27. Keller M, Deveikis A, Cutillar-Garcia M, et al. Pneumococcal and influenza immunization and human immunodeficiency virus load in children. Pediatr Infect Dis J 2000;19:613-8.
28. Glesby MJ, Hoover DR, Farzadegan H, Margolick JB, Saah AJ. The effect of influenza vaccination on human immunodeficiency virus type 1 load: a randomized, double-blind, placebo-controlled study. J Infect Dis 1996;174:1332-6.
29. Sullivan PS, Hanson DL, Dworkin MS, Jones JL, Ward JW. Effect of influenza vaccination on disease progression among HIV-infected persons. AIDS 2000;14:2781-5.
30. Banic S, Koren S, Tomazic J, et al. Influenza vaccination of human immunodeficiency virus 1-infected patients receiving antiretroviral therapy. Acta Virol 2001;45:39-44.
31. Gunthard HF, Wong JK, Spina CA, et al. Effect of influenza vaccination on viral replication and immune response in persons infected with human immunodeficiency virus receiving potent antiretroviral therapy. J Infect Dis 2000;181:522-31.
32. Fuller JD, Craven DE, Steger KA, Cox N, Heeren TC, Chernoff D. Influenza vaccination of human immunodeficiency virus (HIV)-infected adults: impact on plasma levels of HIV type 1 RNA and determinants of antibody response. Clin Infect Dis 1999;28:541-7.
33. Benne CA, Kroon FP, Harmsen M, Tavares L, Kraaijeveld CA, De Jong JC. Comparison of neutralizing and hemagglutination-inhibiting antibody responses to influenza A virus vaccination of human immunodeficiency virus-infected individuals. Clin Diagn Lab Immunol 1998;5:114-7.
34. Nelson KE, Clements ML, Miotti P, Cohn S, Polk BF. The influence of human immunodeficiency virus (HIV) infection on antibody responses to influenza vaccines. Ann Intern Med 1988;109:383-8.
35. Malaspina A, Moir S, Orsega SM, et al. Compromised B cell responses to influenza vaccination in HIV-infected individuals. J Infect Dis 2005;191:1442-50.
36. Weinberg A, Muresan P, Fenton T, et al. Safety and Immunogenicity of Two Doses of Monovalent Pandemic H1N1 (pH1N1) Influenza Vaccine in HIV-Infected Pregnant Women. Presented at: IDSA;August 2010
37. Atashili J, Kalilani L, Adimora AA. Efficacy and clinical effectiveness of influenza vaccines in HIV-infected individuals: a meta-analysis. BMC Infect Dis 2006;6:138.
38. Tasker SA, Treanor JJ, Paxton WB, Wallace MR. Efficacy of influenza vaccination in HIV-infected persons. A randomized, double-blind, placebo-controlled trial. Ann Intern Med 1999;131:430-3.

39. Ranieri R, Veronelli A, Santambrogio C, Pontiroli AE. Impact of influenza vaccine on response to vaccination with pneumococcal vaccine in HIV patients. AIDS Res Hum Retroviruses 2005;21:407-9.
40. Yamanaka H, Teruya K, Tanaka M, et al. Efficacy and immunologic responses to influenza vaccine in HIV-1-infected patients. J Acquir Immune Defic Syndr 2005;39:167-73.
41. Madhi SA, Maskew M, Koen A, et al. Trivalent inactivated influenza vaccine in African adults infected with human immunodeficient virus: double blind, randomized clinical trial of efficacy, immunogenicity, and safety. Clin Infect Dis 2011;52:128-37.
42. Englund JA, Mbawuike IN, Hammill H, Holleman MC, Baxter BD, Glezen WP. Maternal immunization with influenza or tetanus toxoid vaccine for passive antibody protection in young infants. J Infect Dis 1993;168:647-56.
43. Murray DL, Imagawa DT, Okada DM, St Geme JW, Jr. Antibody response to monovalent A/New Jersey/8/76 influenza vaccine in pregnant women. J Clin Microbiol 1979;10:184-7.
44. Munoz FM, Greisinger AJ, Wehmanen OA, et al. Safety of influenza vaccination during pregnancy. Am J Obstet Gynecol 2005;192:1098-106.
45. Black SB, Shinefield HR, France EK, Fireman BH, Platt ST, Shay D. Effectiveness of influenza vaccine during pregnancy in preventing hospitalizations and outpatient visits for respiratory illness in pregnant women and their infants. Am J Perinatol 2004;21:333-9.
46. France EK, Smith-Ray R, McClure D, et al. Impact of maternal influenza vaccination during pregnancy on the incidence of acute respiratory illness visits among infants. Arch Pediatr Adolesc Med 2006;160:1277-83.
47. Demicheli V, Rivetti D, Deeks JJ, Jefferson TO. Vaccines for preventing influenza in healthy adults. Cochrane Database Syst Rev 2004:CD001269.
48. Farquhar C, Nduati R, Haigwood N, et al. High maternal HIV-1 viral load during pregnancy is associated with reduced placental transfer of measles IgG antibody. J Acquir Immune Defic Syndr 2005;40:494-7.
49. Hartter HK, Oyedele OI, Dietz K, Kreis S, Hoffman JP, Muller CP. Placental transfer and decay of maternally acquired antimeasles antibodies in Nigerian children. Pediatr Infect Dis J 2000;19:635-41.
50. Englund JA. The influence of maternal immunization on infant immune responses. J Comp Pathol 2007;137 Suppl 1:S16-9.
51. Weinberg A, Dickover R, Britto P, et al. Continuous improvement in the immune system of HIV-infected children on prolonged antiretroviral therapy. AIDS 2008;22:2267-77.
52. Brundage JF. Interactions between influenza and bacterial respiratory pathogens: implications for pandemic preparedness. Lancet Infect Dis 2006;6:303-12.
53. Ampofo K, Bender J, Sheng X, et al. Seasonal invasive pneumococcal disease in children: role of preceding respiratory viral infection. Pediatrics 2008;122:229-37.
54. O'Brien KL, Walters MI, Sellman J, et al. Severe pneumococcal pneumonia in previously healthy children: the role of preceding influenza infection. Clin Infect Dis 2000;30:784-9.
55. Grabowska K, Hogberg L, Penttinen P, Svensson A, Ekdahl K. Occurrence of invasive pneumococcal disease and number of excess cases due to influenza. BMC Infect Dis 2006;6:58.
56. Sun K, Metzger DW. Inhibition of pulmonary antibacterial defense by interferon-gamma during recovery from influenza infection. Nat Med 2008;14:558-64.
57. McCullers JA. Insights into the interaction between influenza virus and pneumococcus. Clin Microbiol Rev 2006;19:571-82.
58. website. CSF. http://www.cdc.gov/flu/weekly/ Accessed 17 July 2009.
59. Bacterial coinfections in lung tissue specimens from fatal cases of 2009 pandemic influenza A (H1N1) - United States, May-August 2009. MMWR Morb Mortal Wkly Rep 2009;58:1071-4.

60. Surveillance for pediatric deaths associated with 2009 pandemic influenza A (H1N1) virus infection - United States, April-August 2009. MMWR Morb Mortal Wkly Rep 2009;58:941-7.
61. Madhi SA, Klugman KP. A role for Streptococcus pneumoniae in virus-associated pneumonia. Nat Med 2004;10:811-3.
62. Mbelle N, Huebner RE, Wasas AD, Kimura A, Chang I, Klugman KP. Immunogenicity and impact on nasopharyngeal carriage of a nonavalent pneumococcal conjugate vaccine. J Infect Dis 1999;180:1171-6.
63. Hill PC, Cheung YB, Akisanya A, et al. Nasopharyngeal carriage of Streptococcus pneumoniae in Gambian infants: a longitudinal study. Clin Infect Dis 2008;46:807-14.
64. Bryce J, Daelmans B, Dwivedi A, et al. Countdown to 2015 for maternal, newborn, and child survival: the 2008 report on tracking coverage of interventions. Lancet 2008;371:1247-58.
65. Madhi SA, Madhi A, Petersen K, Khoosal M, Klugman KP. Impact of human immunodeficiency virus type 1 infection on the epidemiology and outcome of bacterial meningitis in South African children. Int J Infect Dis 2001;5:119-25.
66. Levin MJ, Song LY, Fenton T, et al. Shedding of live vaccine virus, comparative safety, and influenza-specific antibody responses after administration of live attenuated and inactivated trivalent influenza vaccines to HIV-infected children. Vaccine 2008;26:4210-7.
67. Forrest BD, Pride MW, Dunning AJ, et al. Correlation of cellular immune responses with protection against culture-confirmed influenza virus in young children. Clin Vaccine Immunol 2008;15:1042-53.
68. Weinberg A, Song L, Walker R, et al. Paradoxical Generalized Decrease of Influenza-Specific T-Cell Responses in HIV-Infected Children after Administration of Inactivated Influenza Vaccine and Sporadic Decrease after Live Attenuated Influenza Vaccine. Presented at: XI International Symposium on Respiratory Viral Infection;Bangkok, Thailand, 19-22 February 2009
69. Buchbinder SP, Mehrotra DV, Duerr A, et al. Efficacy assessment of a cell-mediated immunity HIV-1 vaccine (the Step Study): a double-blind, randomised, placebo-controlled, test-of-concept trial. Lancet 2008;372:1881-93.
70. Fiore AE, Shay DK, Broder K, et al. Prevention and control of influenza: recommendations of the Advisory Committee on Immunization Practices (ACIP), 2008. MMWR Recomm Rep 2008;57:1-60.
71. Rowe T, Abernathy RA, Hu-Primmer J, et al. Detection of antibody to avian influenza A (H5N1) virus in human serum by using a combination of serologic assays. J Clin Microbiol 1999;37:937-43.
72. Weinberg A, Louzao R, Mussi-Pinhata MM, et al. Quality assurance program for peripheral blood mononuclear cell cryopreservation. Clin Vaccine Immunol 2007;14:1242-4.
73. Carvalho Mda G, Tondella ML, McCaustland K, et al. Evaluation and improvement of real-time PCR assays targeting lytA, ply, and psaA genes for detection of pneumococcal DNA. J Clin Microbiol 2007;45:2460-6.
74. O'Brien KL, Nohynek H. Report from a WHO working group: standard method for detecting upper respiratory carriage of Streptococcus pneumoniae. Pediatr Infect Dis J 2003;22:133-40.
75. Raisler J, Alexander C, Camp P. Breast-feeding and infant illness: a dose-response relationship? *Am J Public Health.* 1999; 89:25–30.
76. Chantry CJ, Howard CR, Auinger P. Full breastfeeding duration and associated decrease in respiratory tract infection in US children. Pediatrics 2006;117:425-432.
77. Ali HM, Scott R, Toms GL. The effect of foster feeding and bottle feeding expressed breast-milk on the susceptibility of guinea-pig infants to influenza virus. Br J Exp Pathol 1989;70:183-191
78. McMichael, A.J., et al., *Cytotoxic T-cell immunity to influenza.* N Engl J Med, 1983. 309(1): p. 13-7.
79. Lalvani, A., et al., *Rapid effector function in CD8+ memory T cells.* J Exp Med, 1997. 186(6): p. 859-65.

80. Lawrence, C.W., R.M. Ream, and T.J. Braciale, *Frequency, specificity, and sites of expansion of CD8+ T cells during primary pulmonary influenza virus infection.* J Immunol, 2005. 174(9): p. 5332-40.
81. McElhaney, J.E., et al., *Granzyme B: Correlates with protection and enhanced CTL response to influenza vaccination in older adults.* Vaccine, 2009. 27(18): p. 2418-25.
82. McElhaney, J.E., et al., *T cell responses are better correlates of vaccine protection in the elderly.* J Immunol, 2006. 176(10): p. 6333-9.

Maternal Influenza vaccine trial: South Africa: Definitions and criteria to investigate for influenza

DEFINITIONS OF CONFIRMED INFLUENZA, INFLUENZA LIKE ILLNESS AND OTHER REPIRATORY ILLNESSES IN INFANTS

1. **Influenza like illness (ILI) in infants (clinical condition)**

- Fever (≥37.8°C axillary temperature) of acute onset (<7 days) without an apparent source, as documented by parent/ caregiver/ study staff
  - No source means that there is no apparent cause for the fever, such as soft tissue infection, although generalized symptoms such as irritability, loss of appetite, and/or lethargy may be present;

  OR

- Fever (documented as ≥37.8°C and/ or mother's perception that infant feverish/ hot) plus at least one sign/ symptom of acute respiratory infection WITHIN THE PAST 72 HOURS

  OR

- At least TWO signs/ symptoms of ARTI WITHIN THE PAST 72 HOURS
  - Tachypnoea (RR≥60 breaths /min in infant 0-2 months of age; RR≥50 br/min in infant 2-12 months of age)
  - Difficulty breathing (reported by mother: noisy/ interrupted/ irregular/ or fast)
  - Coughing
  - Wheezing
  - Runny or congested nose
  - Cyanosis/ O_2 saturation < 90% (if available)
  - Chest wall indrawing
  - Grunting on expiration
  - Pus draining from ear

2. **Laboratory Confirmed Influenza (LCI)**

- Infant with laboratory confirmed influenza (PCR from NASAL SAMPLE positive for Influenza A or B) and at least one of the following: Fever, cough, runny nose, wheezing, difficulty breathing, tachypnoea.

3. **Pneumonia (as defined by WHO)**

- Infant with cough or difficulty breathing

AND

- Tachypnoea (RR≥60 breaths/min in infant 0-2 months of age; RR≥50 breaths /min in infant 2-12 months of age)

4. **WHO criteria for Severe pneumonia or very severe disease**

- Infant with cough or difficulty breathing

  AND
  - Chest wall indrawing in a calm child

  OR
  - Stridor in a calm child

  OR
  - Any danger sign
    - If <2 months: unable to drink/nurse, vomits everything, convulsions, tachypnoea (RR>60br/min), severe chest wall indrawing, lethargy or unconsciousness, fever ≥37.5°C/ hypothermia <35.5°C
    - If ≥2 months: unable to drink/nurse, vomits everything, convulsions, lethargy or unconsciousness

INVESTIGATIONS FOR INFLUENZA TO BE DONE ON:

1. All Infants presenting to clinic for unsolicited illness visits with at least one respiratory symptom/ fever (documented ≥37.8°C or reported as feverish/ hot by mother) documented in previous 7 days, irrespective of whether or not they fulfill ILI definition
2. All infants fulfilling ILI definition (which will include infants with WHO defined pneumonia/ severe pneumonia/ very severe disease)
3. All infants hospitalized for ANY respiratory illness of presumed infectious origin (excluding neonates admitted at birth with TTN/ meconium aspiration).
4. Any infant <1 month of age with a history of apnoea attack in past 72 hours.

Term	Definition
Difficulty breathing	Mother's report that her child is having trouble breathing; she may describe the breathing pattern as rapid, noisy, interrupted, or irregular
*Fever	Any of the following:<br>• Mother's perception that child had a fever during the previous 24 hours<br>• Mother measured the child's temperature as >38°C during the previous 24 hrs<br>• Clinician or study staff measure the child's temperature to be >38°C
Lethargy	Child is difficult to awaken when the mother speaks, gently shakes the child, or claps her hands; Is drowsy and uninterested in his/her surroundings
Lower chest wall indrawing	The lower chest wall (lower ribs) goes IN when the child breathes IN. The indrawing must be present when the child is calm, it must be clearly visible, and present all the time. If <u>only</u> the soft tissue between the ribs goes in when the child breathes in, this is not chest indrawing.
Rapid breathing	• < 2 months of age: ≥60 breaths per minute<br>• 2 to 11 months: ≥50 breaths per minute
Stridor	A harsh noise when the child breathes IN, sometimes only heard by listening near the child's mouth. The stridor must be present when the child is calm.
Wheeze	A musical sound when the child breathes OUT. Should not confuse with a wet noise when the nose is blocked or there is mucus in the airway that clears when the nose is cleaned and the child coughs.

DEFINITIONS OF CONFIRMED INFLUENZA, INFLUENZA LIKE ILLNESS AND OTHER REPIRATORY ILLNESSES IN MATERNAL PARTICIPANTS (ADULTS)

1. **Adult Case definition of ILI: (symptom onset within past 7 days)**

- Fever (≥38°C on oral measurements) or chills/ rigors or feeling feverish in past 7 days

  AND
  - Cough/ sore throat/ Pharyngitis

  OR
  - Muscle, joint or headache

  OR
  - Feeling short of breath, had difficulty breathing or chest pain while breathing

  AND
  - Absence of other diagnosis

2. **Laboratory Confirmed Influenza (LCI)**
- Adult participant with laboratory confirmed influenza (PCR from NP/ OP swab positive for Influenza A or B)

3. **WHO definition of Severe Acute Respiratory Infection (SARI)**

   Sudden onset of fever over 38°C, **AND**
- Cough or sore throat**, AND**
- Shortness of breath or difficulty breathing**, AND**
- Requiring hospital admission

INVESTIGATIONS FOR INFLUENZA TO BE DONE ON:
1. All maternal participants fulfilling ILI definition (which will include participants with WHO defined SARI)
2. All adult participants hospitalized for respiratory illness of presumed infectious origin
3. All participants attending clinic for unsolicited respiratory illness visits with at least one respiratory symptom/ fever documented in previous 7 days, irrespective of whether or not they fulfill ILI definition

Other definitions:

1. Low birth weight: <2500g at birth
2. Premature delivery/ infant: <37 completed weeks gestation, by best estimate (using SOP-LMP, early sonar, SFH serial measurements)
